# Discovery of Novel Na_v_1.7-Selective Inhibitors with the 1*H*-Indole-3-Propionamide Scaffold for Effective Pain Relief

**DOI:** 10.34133/research.0599

**Published:** 2025-01-29

**Authors:** Gaoang Wang, Hang Wu, Yingying Wang, Xiangying Liu, Shuijiao Peng, Wenxing Wang, Meijing Wu, Yifei Liu, Ercheng Wang, Zhe Wang, Lei Xu, Xiaojian Wang, Wei Yang, Haiyi Chen, Xi Zhou, Tingjun Hou

**Affiliations:** ^1^ College of Pharmaceutical Sciences, Zhejiang University, Hangzhou, Zhejiang 310058, China.; ^2^ The National and Local Joint Engineering Laboratory of Animal Peptide Drug Development, College of Life Sciences, and Peptide and Small Molecule Drug R&D Platform, Furong Laboratory, Hunan Normal University, Changsha 410081, Hunan, China.; ^3^ Zhejiang University School of Medicine, Hangzhou 310000, Zhejiang, China.; ^4^ State Key Laboratory of Bioactive Substance and Function of Natural Medicines and Beijing Key Laboratory of Active Substances Discovery and Druggability Evaluation, Institute of Materia Medica, Chinese Academy of Medical Sciences and Peking Union Medical College, Beijing 100050, China.; ^5^ Zhejiang Laboratory, Hangzhou 311100, Zhejiang, China.; ^6^ School of Pharmacy, Hangzhou Normal University, Hangzhou 311121, Zhejiang, China.; ^7^ Institute of Bioinformatics and Medical Engineering, School of Electrical and Information Engineering, Jiangsu University of Technology, Changzhou 213001, Jiangsu, China.

## Abstract

Na_v_1.7 is considered a promising target for developing next-generation analgesic drugs, given its critical role in human pain pathologies. Although most reported inhibitors with strong in vitro activity and high selectivity share the aryl sulfonamide scaffold, they failed to demonstrate marked clinical efficacy. Therefore, exploring new Na_v_1.7-selective antagonists is quite urgent to the development of next-generation analgesic drugs. Here, we report a highly effective 1*H*-indole-3-propionamide inhibitor, WN2, identified through an integrated drug discovery strategy. Notably, the structure of WN2 is quite different from previously reported aryl sulfonamide inhibitors. Molecular dynamics simulations and experimental findings reveal that the R configuration of WN2 (WN2-R) is the preferred form (IC_50_ = 24.7 ± 9.4 nM) within the VSDIV pocket of Na_v_1.7. WN2-R exhibits impressive analgesic effects in acute and chronic inflammatory pain, as well as neuropathic pain models in mice. Additionally, it displays favorable subtype selectivity and positive drug safety in acute toxicity studies. Pharmacokinetic studies indicate that WN2-R has high bioavailability (*F* = 20.29%), highlighting its considerable potential for drug development. Our study establishes WN2-R as a novel Na_v_1.7-selective inhibitor with a unique structural scaffold, offering a promising candidate for the next generation of analgesic drugs.

## Introduction

Voltage-gated sodium channels (VGSCs/Na_v_s), as key components of voltage-gated ion channels (VGICs), are crucial for generating and transmitting electrical signals within cells [1]. In mammals, each Na_v_ consists of a pore-forming α subunit (~2,000 amino acids) and auxiliary β subunits [2,3]. The α subunit consists of 4 domains (DI to DIV), with each domain containing 6 transmembrane α-helices (S1 to S6). The segments S1 to S4 constitute the voltage-sensing domain (VSD), which is responsible for sensing changes in external voltage. The central pore and selectivity filter are formed by the S5 to S6 segments and the extracellular pore loops (P-loops), which control ion selectivity and permeation. Structural biology studies have identified at least 3 physiological states in VGSCs: activated (open), inactivated (closed), and resting (closed) [4]. Nowadays, 9 Na_v_ subtypes (Na_v_1.1 to Na_v_1.9) have been discovered in humans, each exhibiting distinct tissue distributions and unique functions [5]. Briefly, Na_v_1.1, Na_v_1.2, Na_v_1.3, and Na_v_1.6 are mainly expressed in the central nervous system (CNS), Na_v_1.4 is mainly expressed in skeletal muscle, Na_v_1.5 is crucial for heart muscle regulation, and Na_v_1.7, Na_v_1.8, and Na_v_1.9 play a role in regulating the function of the peripheral nervous system (PNS), playing pivotal roles in pain signaling. Notably, gain-of-function mutations in Nav1.7 have been linked to hereditary pain disorders like inherited erythromelalgia and paroxysmal extreme pain disorder, while loss-of-function mutations result in congenital inability to experience pain. Therefore, Na_v_1.7 serves as a prospective target for the development of innovative nonopioid pain therapeutics.

Over the years, a number of traditional Na_v_s inhibitors have been used as pain relievers. One example is the local anesthetic lidocaine, which targets the local anesthetic binding site (site 8) of Na_v_s [5] for pain management. However, one important limitation of these drugs is their inability to selectively target specific subtypes, as they act on other subtypes besides Na_v_1.7 (particularly Na_v_1.5), leading to adverse side effects including cardiac arrhythmias, liver injury, headaches, and leukopenia [6–9]. Hence, the focus has shifted to developing selective Na_v_1.7 inhibitors for the next generation of Na_v_s analgesics. In 2013, Pfizer developed PF-05089771, a selective aryl sulfonamide small-molecule inhibitor targeting the VSD of DIV (VSDIV) of Na_v_1.7 [1,10]. Since then, a series of similar selective inhibitors were developed, most sharing an aryl sulfonamide-like structure and targeting the VSDIV pocket. Unfortunately, PF-05089771 failed to demonstrate marked therapeutic benefits in phase II clinical trials compared to placebo [11,12]. Besides, other homologs did not show expected clinical efficacy. Despite exhibiting strong activity (highest activity < 1 nM) and high selectivity (over 1,000-fold), these Na_v_1.7-selective inhibitors fell short as potential next-generation pain medications. Therefore, abandoning the sulfonamides core and exploring new Na_v_1.7-selective antagonists represents a crucial step toward developing next-generation Na_v_s analgesics.

In this study, an integrated drug discovery strategy by combining structure-based virtual screening (SBVS), molecular dynamics (MD) simulation, computational structural optimization, electrophysiological test, and mice pain model was employed to develop selective Na_v_1.7 inhibitors. By screening over 1.7 million compounds, a promising series of 1*H*-indole-3-propionamide inhibitors were initially identified. Among these, compound WN2 stood out due to its novel structure and remarkable activity. Extensive MD simulation over 4 μs and the electronic circular dichroic (ECD) spectrum method confirmed WN2-R as the dominant active configuration (IC_50_ = 24.7 ± 9.4 nM). The MD simulations and point mutation experiments verified that WN2-R bound to the intended VSDIV pocket. The current-clamp recording results showed that WN2-R could decrease the membrane excitability of dorsal root ganglia (DRG) neurons, thereby inhibiting the generation and propagation of pain signals. Furthermore, we evaluated the effectiveness of WN2-R through diverse mice pain models, including acute and chronic inflammatory pain, and neuropathic pain models, using various routes of administration, including intragastric (i.g.) administration, intraperitoneal (i.p.) injection, and intramuscular (i.m.) injection. WN2-R demonstrated superior performance across these pain models, comparable or even better when compared to PF-05089771. Furthermore, WN2-R exhibited satisfactory selectivity, toxicity, and bioavailability. Collectively, these results underscored the obvious potential of WN2-R as a potential candidate for the development of a novel generation of analgesics.

## Results

### Identification of WN2 as a novel Na_v_1.7 inhibitor

**Fig. 1.**
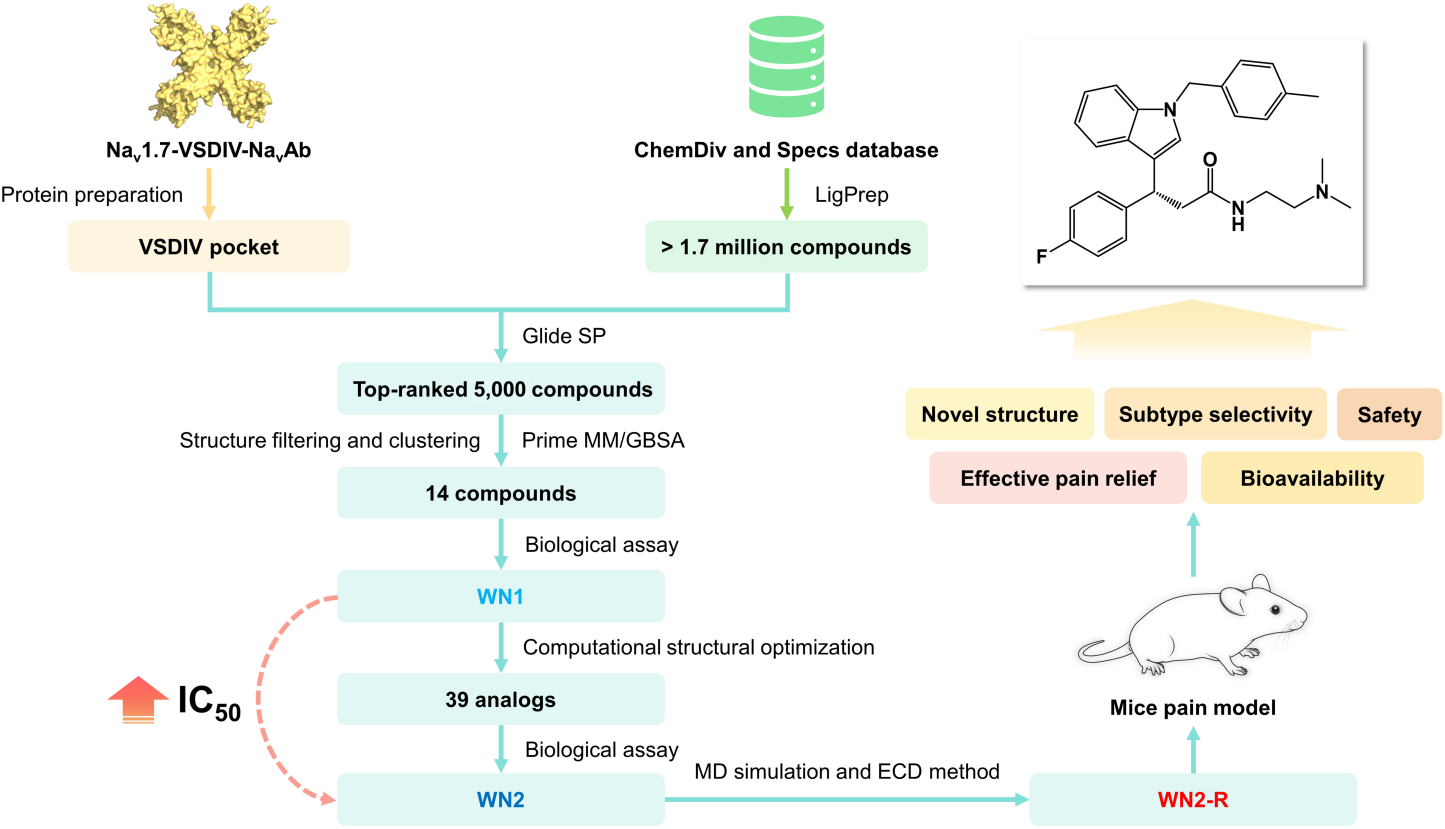
The workflow of the integrated drug discovery strategy used in this study.

**Fig. 2.**
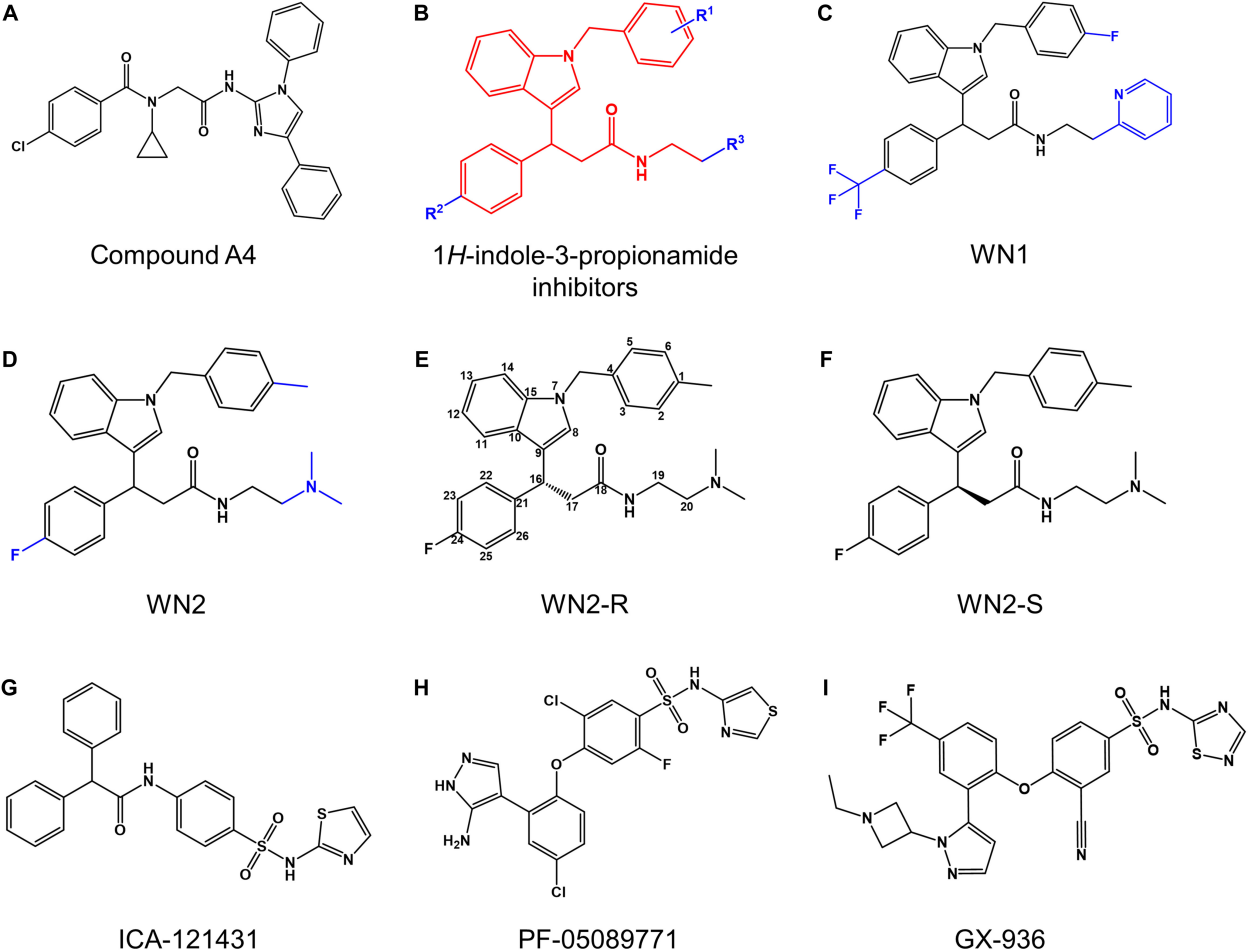
The chemical structures of the compounds mentioned in our study. (A) The chemical structure of compound A4; (B) The structural scaffold of WN1; (C) The chemical structure of WN1; (D) The chemical structure of WN2; (E) The chemical structure of WN2-R with carbon atoms numbered; (F) The chemical structure of WN2-S; (G) The chemical structure of ICA-121431; (H) The chemical structure of PF-05089771; (I) The chemical structure of GX-936.

**Fig. 3.**
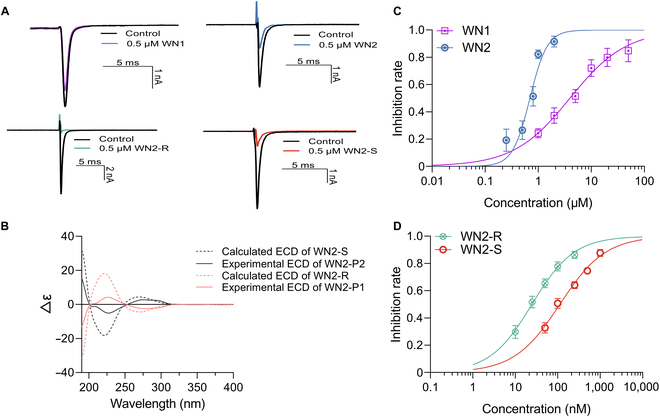
The inherent relevance between the antagonistic activity and the chirality of WN2. (A) Example traces illustrating the inhibition of 0.5 μM compounds on Na_v_1.7 currents elicited by voltage protocols as shown (*n* = 3 to 7). (B) The relationship between the difference in absorption coefficients (Δε) of compounds and the variation of wavelength (190 to 400 nm), indicating that WN2-P1 was the R configuration. (C and D) Concentration–response relationships of compounds inhibiting Na_v_1.7 at −80 mV holding, and the IC_50_ values were determined as 3.96 ± 0.14 μM (WN1), 0.73 ± 0.12 μM (WN2), 24.7 ± 9.4 nM (WN2-R), and 112.9 ± 36.9 nM (WN2-S), respectively (*n* = 5 to 7).

The integrated drug discovery strategy used in this work is outlined in Fig. 1. Over 1.7 million compounds were screened, and a total of 14 candidate compounds were identified (Table S1). To validate their activity, inhibition assays were conducted at a concentration of 10 μM. Three compounds demonstrated inhibition rates exceeding 50%, and notably compound A4 (Fig. 2A) exhibited the highest inhibition rate at 76.38%. However, it was observed that compound A4 shared an identical structural scaffold with compound **2,** as previously reported by Chandra et al. [13,14], and this class of compounds had already been protected by patents. This attested to the soundness of our screening strategy, demonstrating its efficacy in discovering potential bioactive compounds. Compounds A6 and A7 possess a novel structural scaffold distinct from aryl sulfonamide compounds. Therefore, A6, known as WN1 (Fig. 2C), was chosen for further activity analysis. The half-maximal inhibitory concentration (IC_50_) for Na_v_1.7 inhibition was determined to be 3.96 ± 0.14 μM (Fig. 3C).

Although WN1 showed promising activity, there was still potential for further enhancement. Therefore, we employed computational structural optimization methods to search for analogs with higher activity. Two different search methods were employed based on the structural scaffold (Fig. 2B) or the entire 2-dimensional (2D) structure of WN1. After conducting a thorough structure–activity relationship (SAR) analysis, a total of 39 analogs were identified and shown in Table S2. These compounds were then subjected to inhibition activity testing under identical experimental conditions. Fortunately, compound B10, also known as WN2 (Fig. 2D), exhibited better activity than WN1, with an IC_50_ of 0.73 ± 0.12 μM (Fig. 3C). As a result, we successfully identified a novel Na_v_1.7 inhibitor, WN2, which possesses a unique structural divergence from aryl sulfonamide compounds.

### The R configuration of WN2 was the primary active form

**Fig. 4.**
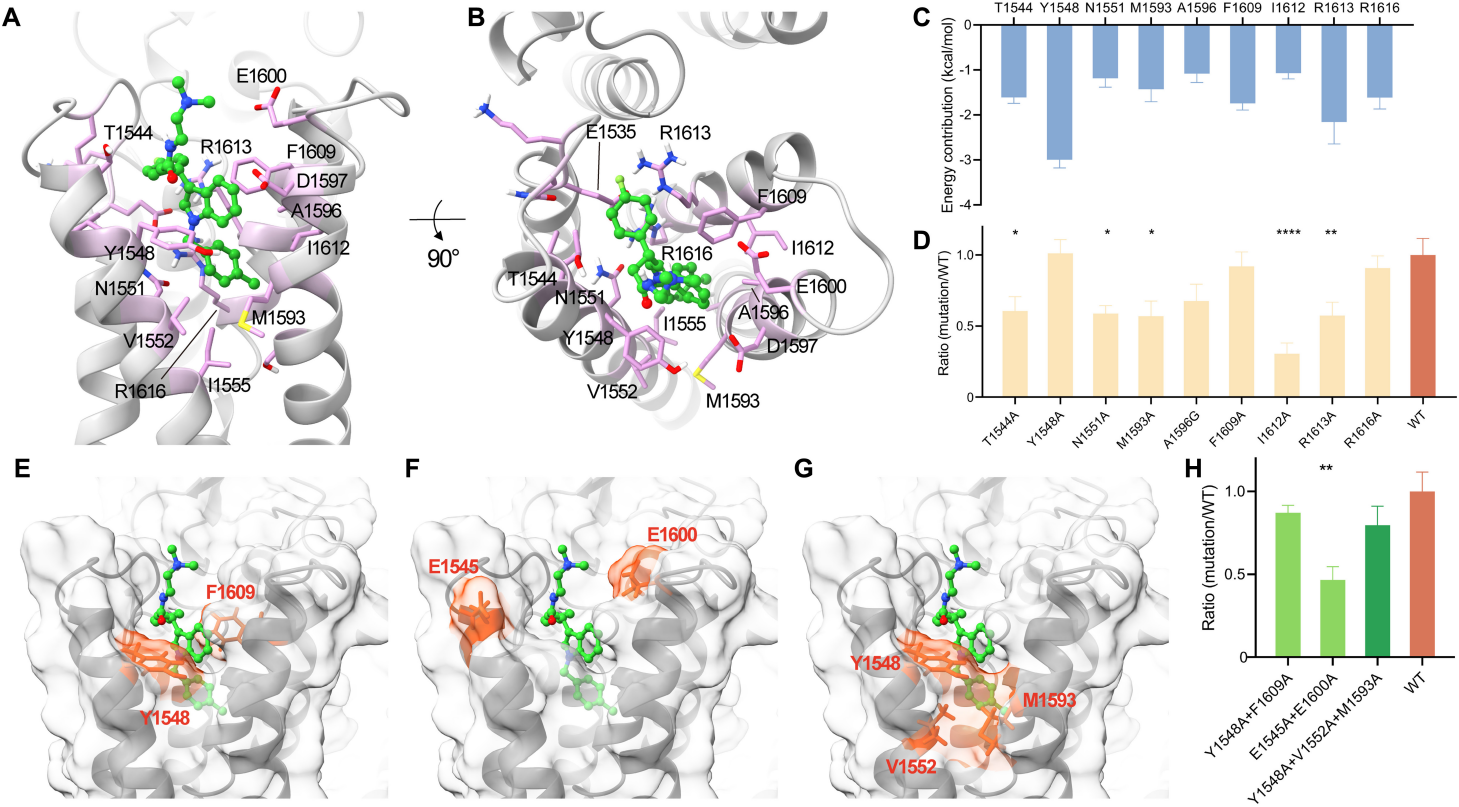
The binding conformation of WN2-R in the VSDIV pocket predicted by the MD simulations and point mutation analysis. (A and B) The side view and top view of the representative binding conformation of WN2-R in the VSDIV pocket predicted by the MD simulations. (C) The important residues for ligand binding calculated by the VAD-MM/GBSA energy decomposition. (D and H) The result of the single and multiple site-directed mutagenesis of WN2-R at 1 μM (*P < 0.05; ** *P* < 0.01; ****P < 0.0001); WT: wild type. (E to G) The amino acid position of multiple point mutations in the binding conformation of WN2-R. The ligand was shown as stick–ball models, and the amino acids of multiple point mutations were shown as transparent surface.

Since WN2 has a chirality center (16th carbon atom), we sought to explore a potential connection between its antagonistic activity and chirality. To clarify this issue, we initially used computational methods for prediction. Both the R and S configurations of WN2 (Fig. 2E and F) were docked into the VSDIV pocket of Na_v_1.7 [Protein Data Bank (PDB) code: 6J8J] using the Induced Fit Docking (IFD) method. After obtaining complex structures for both configurations, a 500-ns MD simulation was performed for each system. To ensure the reliability of the simulation results, 4 replicas were launched for each system. The observations revealed that the R configuration stably bound to the VSDIV pocket across all 4 trajectories (the representative binding conformation shown in Fig. 4A and B), while the S configuration showed continuous conformational fluctuation and even a tendency to dissociation. To further analyze the interactions, we calculated the binding affinity (kcal/mol) using VAD-MM/GBSA and measured ligand fluctuation through root-mean-square fluctuation (RMSF) values. The results (Table 1) showed that WN2-R exhibited an average binding affinity of −49.94 ± 2.67 kcal/mol with an RMSF average of 2.35 ± 0.58 Å. In contrast, WN2-S exhibited a weaker average binding affinity of −32.15 ± 10.39 kcal/mol and a markedly higher RMSF average of 11.86 ± 12.31 Å. Both indicators suggested a stronger binding between WN2-R and the VSDIV pocket. Therefore, we supposed that the R configuration of WN2 was primarily responsible for its activity.

**Table 1.** The binding affinity (kcal/mol) calculated by VAD-MM/GBSA and ligand fluctuation reflected by the RMSF values for the MD trajectories

	Compound	Traj 1	Traj 2	Traj 3	Traj 4	Average
Binding free energy	WN2-R	−47.77	−53.57	−50.31	−48.12	−49.94 ± 2.67
	WN2-S	−31.62	−20.84	−30.14	−45.99	−32.15 ± 10.39
RMSF	WN2-R	2.36	2.50	1.56	2.96	2.35 ± 0.58
	WN2-S	8.60	5.51	30.03	3.29	11.86 ± 12.31

To validate the theoretical calculation, we chemically synthesized WN2 and conducted chiral separation, yielding 2 enantiomerically pure samples, P1 and P2. Both samples were tested for their activity under identical conditions. Surprisingly, P1 exhibited an IC_50_ of 24.7 ± 9.4 nM, while P2 displayed an IC_50_ of 112.9 ± 36.9 nM (Fig. 3D). The activity of P1 was 4.57 times higher than that of P2, revealing a marked difference in activity between the enantiomers. To confirm the chirality of P1, the ECD method was employed for chiral analysis. The result confirmed that P1 corresponded to the R configuration, while P2 was of the S configuration (Fig. 3B). The experimental group curve aligned closely with the theoretical group curve. Collectively, the MD simulation results and ECD identification consistently point out the predominance of WN2-R in exerting antagonistic activity. Interestingly, the racemic sample of WN2 did not exhibit activity intermediate between P1 and P2; instead, its activity was inferior to both enantiomers. This might be attributed to the potential interference or mutual influence between the different chiral forms of the compound.

### The targeting verification of WN2-R

From the representative binding conformation of WN2-R depicted in Fig. 4A and B, it was interesting to observe that the ligand was surrounded by hydrophobic residues, lacking any specific polar interactions in this particular conformation. To validate whether WN2-R truly bound to the Na_v_1.7 VSDIV pocket, a combination of MD simulations and site-directed mutagenesis analysis was utilized. Initially, a VAD-MM/GBSA energy decomposition was utilized, and the results revealed several important residues that markedly contribute to ligand binding (energy contribution < −1.0 kcal/mol, Fig. 4C). Unsurprisingly, nonpolar residues such as Y1548, M1593, A1596, F1609, and I1612 collectively made substantial contributions to the binding free energy of WN2-R. Additionally, R1613 and R1616 interacted with WN2-R through cation–π conjugations.

The amino acids depicted in Fig. 4C were individually mutated to alanine or glycine to perform single site-directed mutagenesis. Subsequently, the inhibition rates of these mutations against WN2-R were measured at a concentration of 1 μM (Fig. 4D). The result showed that mutations of several residues with marked energy contributions led to a decrease in the antagonistic activity of WN2-R, including T1544A (−1.61 kcal/mol, 60.7% of WT), N1551A (−1.18 kcal/mol, 58.9% of WT), M1593A (−1.43 kcal/mol, 57.1% of WT), I1612A (−1.07 kcal/mol, 30.6% of WT), and R1613A (−2.16 kcal/mol, 57.4% of WT). Additionally, A1596G also exerted an influence on the compound’s activity. These results strongly suggest that WN2-R bound at the supposed VSDIV binding site, validating our MD prediction. While single site-directed mutagenesis did affect the antagonistic activity of WN2-R, it did so to a limited extent, as the presumed induced-fit effect typically arises from the concerted movement of multiple residues. However, unexpectedly, the introduction of Y1548A and F1609A had minimal impact on the antagonistic activity of WN2-R.

To further investigate the roles of these residues in WN2-R binding, 3 combinations of double or triple site-directed mutagenesis were introduced, including Y1548A/F1609A (to investigate the function of phenyl ring-containing residues surrounding WN2-R, Fig. 4E), E1545A/E1600A (to examine the importance of negatively charged residues at the binding site entrance, Fig. 4F), and Y1548A/V1552A/M1593A (to understand the role of the outer hydrophobic wall formed by these residues in ligand binding, Fig. 4G). As shown in Fig. 4H, the combined mutation of E1545A and E1600A had an inhibitory effect on WN2-R activity, suggesting that these 2 residues should be involved in the entry of WN2-R into the binding pocket, while the introduction of Y1548A/F1609A or Y1548A/V1552A/M1593A had minimal effects on WN2-R activity, consistent with the findings from the single mutation results. Notably, the introduction of I1612A had the most profound effect on WN2-R activity among all single site-directed mutations, suggesting that the strength of WN2-R binding was more reliant on the inner hydrophobic residues of the binding pocket, even though the external hydrophobic residues contributed more to the binding free energy.

### WN2-R specifically inhibited sodium channels and decreased the membrane excitability of DRG neurons

**Fig. 5.**
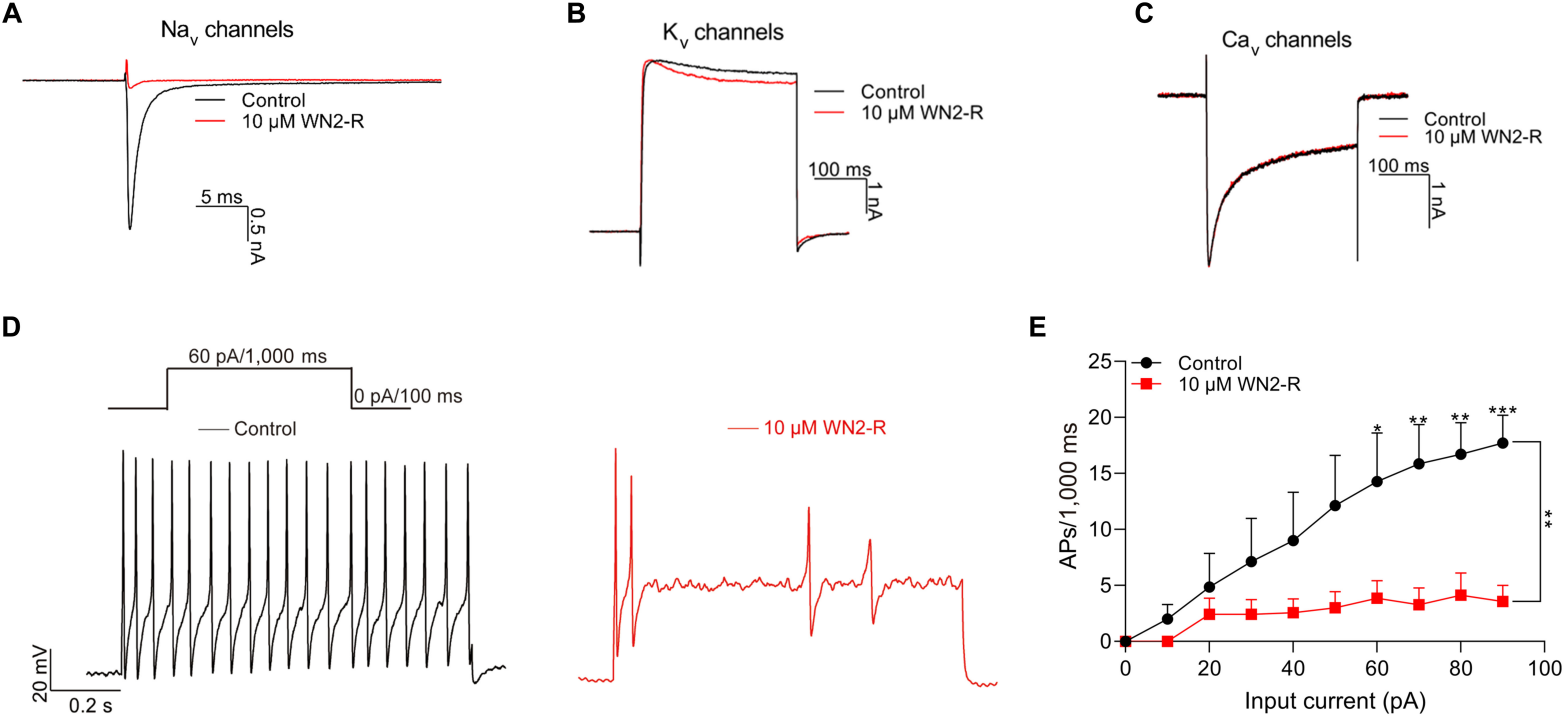
The effect of WN2-R on membrane excitability and influence on Na_v_, K_v_, and Ca_v_ channels in DRG neurons. (A to C) Representative current traces from mouse DRG neurons expressing native Na_v_ (*n* = 4), K_v_ (*n* = 4), or Ca_v_ (*n* = 4) channels currents, recorded before and after treatment with 10 μM WN2-R (red). (D) AP traces recorded from a representative mouse DRG neurons before (black) and after (red) the application of 10 μM WN2-R. (E) Statistics plots showed significant decreases in AP spike number in the presence of 10 μM WN2-R (*n* = 7; *P < 0.05; **P < 0.01; ***P < 0.001).

To investigate the mechanism of action of WN2-R and facilitate animal model experiments, we used voltage-clamp recording to test the effects of WN2-R on the native Na^+^-, K^+^-, and Ca^2+^-mediated currents in DRG neurons. As shown in Fig. 5A to C, at a concentration of 10 μM, WN2-R completely inhibited the native Na^+^ current while having almost no effect on the native K^+^ and Ca^2+^ currents. These results indicated that WN2-R primarily exerted its effect by inhibiting sodium channels, with negligible impact on potassium and calcium channels. Furthermore, the effects of WN2-R on membrane excitability were examined in small (<30 μm) DRG neurons from mice using current-clamp recordings. As shown in Fig. 5D and E, WN2-R markedly reduced the action potential (AP) firing of these neurons in response to depolarizing currents. These results suggested that WN2-R could decrease the membrane excitability of small DRG neurons, thereby blocking pain signal transmission and exerting an analgesic effect. In summary, WN2-R specifically inhibited sodium channels, markedly reduced the membrane excitability of DRG neurons, and showed potential as an analgesic agent.

### WN2-R reduced acute inflammatory pain in mice

**Fig. 6.**
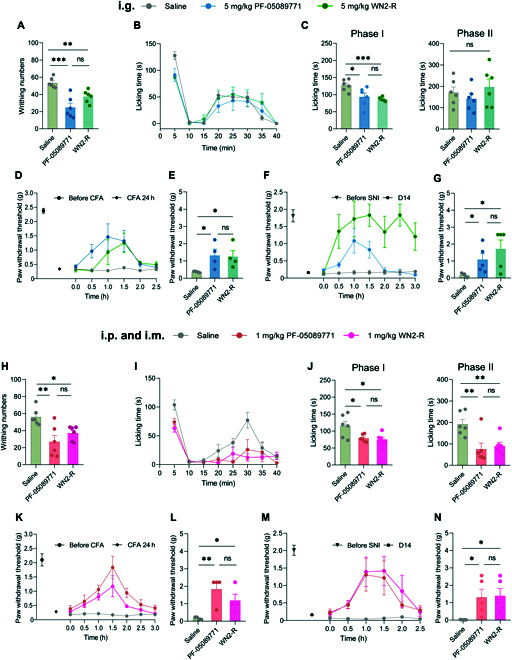
Profiling the analgesic potential of WN2-R in mice models of acute and chronic inflammatory and neuropathic pain. The legend in the upper panel denotes the various experimental groups in these behavioral tests. (A and H) Acetic acid-induced writhing test (i.g. and i.p.), with WN2-R and PF-05089771 notably reducing the writhing number (*n* = 6). (B, C, I, and J) Formalin-induced inflammation pain model (i.g. and i.m.): (B and I) time course of licking times of mice in each group; (C and J) statistics of the total licking times in phase I (left) and phase II (right) for each group (*n* = 6). (D, K, E, and L) CFA-induced chronic pain model (i.g. and i.m.): (D and K) time course of PWTs of mice in each group; right panel, 24 h post CFA treatment, the PWTs in mice were markedly reduced, confirming the successful establishment of the model (*n* = 24); (E and L) statistics of the PWTs 1.5 h post drug application (*n* = 4). (F, M, G, and N) SNI pain model (i.g. and i.m.): (F and M) time course of PWTs of mice in each group; left panel, PWTs of mice before and after SNI (14 days following the surgery), showing successful establishment of the model (*n* = 30); (G and N) statistics of the PWTs at 1 h post drug application (*n* = 5). Note that statistical differences between the drug groups and saline control were assessed by one-way ANOVA with post-hoc analysis using the Dunnett method, and comparison between the drug groups and saline groups was performed with unpaired *t* test (^*^P < 0.05; ^**^P < 0.01; ^***^P < 0.001; ns, not significantly different). The experimenters were blinded to the treatment condition.

Acute pain was a common type of pain with considerable value for drug development. Here, 2 acute inflammatory pain models were used to verify the acute analgesic effect of WN2-R. One such model, the acetic acid writhing pain model in mice, is a well-established acute visceral pain model. It simulates clinically relevant intestinal discomfort by stimulating the abdominal mucosa and intestine with acetic acid and has been extensively used to screen analgesic or anti-inflammatory drugs [15,16]. Based on this model, we evaluated the acute analgesic effect of WN2-R using 2 drug delivery methods, i.g. and i.p. administration (*n* = 6). Both methods clearly showed a significant decrease in writhing behavior in the WN2-R group compared to the saline group, as evidenced by statistically significant differences (Fig. 6A and H). Furthermore, the analgesic effect of WN2-R at the same dose was similar to that of the positive control drug PF-05089771, with no significant difference between these two. After normalizing the number of writhing, the analgesic rates of the WN2-R group are 30.0% and 34.1% for i.g. and i.p., respectively, compared to 53.5% and 51.9% for PF-05089771. A comparison of the 2 drug delivery methods indicated that WN2-R had good oral efficacy. These results strongly suggested that WN2-R had an acute analgesic effect in the acetic acid writhing pain model of mice.

The formalin-induced acute inflammatory pain model in mice, achieved through the plantar injection of formalin aqueous solution, is another widely used model for acute inflammatory pain. This model comprises 2 stages: an initial phase (0 to 15 min) where pain is triggered by formalin's direct stimulation of nociceptive receptors on sensory neurons, followed by a second phase (15 to 40 min) characterized by persistent inflammatory pain due to tissue damage [17]. Similarly, both i.g. and i.m. methods were used to evaluate the effect of WN2-R in this model (*n* = 6) (Fig. 6B and I). During phase I, both the WN2-R and PF-05089771 groups exhibited significantly reduced licking time compared to the saline control group, regardless of the administration route (i.g. or i.m.), with statistically significant differences (Fig. 6C and J). The effects of WN2-R were comparable to those of PF-05089771. However, during phase II, WN2-R failed to significantly reduce licking time when administered intragastrically (Fig. 6C). However, i.m. administration of both WN2-R and PF-05089771 significantly reduced licking time compared to the saline group (Fig. 6J). These findings were consistent across repeated experiments. These results suggested that WN2-R exhibited rapid analgesic effects during phase I of the model, regardless of whether it was administered i.m. or i.g., effectively relieving pain reactions caused by direct formalin stimulation. However, in phase II, characterized by persistent inflammatory pain, WN2-R only exhibited efficacy under i.m. conditions. This observation might be attributed to its limited oral bioavailability in this particular model, as evidenced by the decreased effectiveness of the positive control drug PF-05089771. Additionally, the pain experienced during phase II was primarily caused by inflammation resulting from tissue damage, suggesting that WN2-R might be more effective against direct stimuli than against inflammatory processes. This might also suggest that there was room for further optimizing the indications of WN2-R. Overall, these results from both acute pain models in mice highlighted the promising acute analgesic effects exhibited by WN2-R.

### Analgesic effect of WN2-R on chronic inflammatory pain

The chronic pain model of mice was often used to simulate persistent pain in humans. In this model, chronic persistent pain was induced by injecting complete Freund’s adjuvant (CFA) into the right hind foot of mice [18]. Following CFA administration, the paw withdrawal thresholds (PWTs) of mice significantly dropped from 2.38 to 0.34 g under i.g. (*n* = 12) and from 2.11 to 0.34 g under i.m. (*n* = 12), confirming the successful establishment of the model (Fig. 6D and K). The chronic analgesic potential of WN2-R was evaluated using both i.g. and i.m. methods (*n* = 4). When administered by i.g., the PWTs of the WN2-R group and the PF-05089771 group reached their peak at 1.5 and 1 h after administration, respectively, and subsequently declined to the same level as the saline group at 2.5 h (Fig. 6D). At 1.5 h, the PWT values for the 2 groups were 1.25 and 1.32 g, respectively (Fig. 6E), both significantly higher than those of the saline group. When administered i.m., both the WN2-R and PF-05089771 groups reached peak PWTs at 1.5 h, and declined to the saline group level at 3 h (Fig. 6K). The PWTs of the WN2-R and PF-05089771 groups at 1.5 h were 1.17 and 1.83 g, respectively, which were significantly higher than those of the saline group (Fig. 6L) and basically the same as the values measured under i.g. conditions. Collectively, these results demonstrated the chronic analgesic properties of WN2-R in the CFA inflammatory pain model of mice.

### Antiallodynic effect of WN2-R on the spared nerve injury model

Peripheral nerve injury models were developed to investigate the mechanisms of neuropathic pain. One such model, the spared nerve injury (SNI) neuropathic pain model in mice, involved ligating and transecting the infraorbital and tibial nerves within the trigeminal nerve, while leaving the sural nerve untouched. This resulted in persistent hypersensitivity to mechanical stimuli on the ipsilateral hind paw, lasting for at least 4 weeks, making it a reliable and frequently used model for neuropathic pain research [19,20]. Fourteen days after surgery, the PWTs of the SNI group significantly decreased from 1.81 to 0.16 g under i.g. (*n* = 15) and from 2.01 to 0.16 g under i.m. (*n* = 15) compared to before surgery, indicating hypersensitivity and successful modeling (Fig. 6F and M). To evaluate the neuroanalgesic potential of WN2-R in this model, both i.g. and i.m. routes were used to evaluate the neuroanalgesic effect of WN2-R in this model (*n* = 5). Under i.g. conditions, WN2-R exhibited rapid onset and significant efficacy after 1 h, lasting for at least 2 h, with a PWTs of 1.72 g. In contrast, PF-05089771 reached its peak at 1 h (PWTs = 1.08 g) but then rapidly lost its efficacy, resembling the saline control group after 1 h. Throughout the experiment, the PWTs of the WN2-R group remained consistently higher than that of the PF-05089771 group (Fig. 6F and G). Under i.m. conditions, both WN2-R and PF-05089771 exhibited rapid onset of analgesic effects, reaching peak efficacy after 1 h with PWTs of 1.39 and 1.30 g, respectively. Subsequently, the efficacy of both drugs gradually declined, converging with the saline group after 3 h. Throughout the entire dosing period, the PWTs of the WN2-R group were slightly higher than that of the PF-05089771 group (Fig. 6M and N). These results demonstrate that WN2-R effectively alleviated SNI neuropathic pain in mice and outperformed the positive control drug PF-05089771, exhibiting robust neuroanalgesic properties.

### The selectivity, safety, and bioavailability of WN2-R

**Fig. 7.**
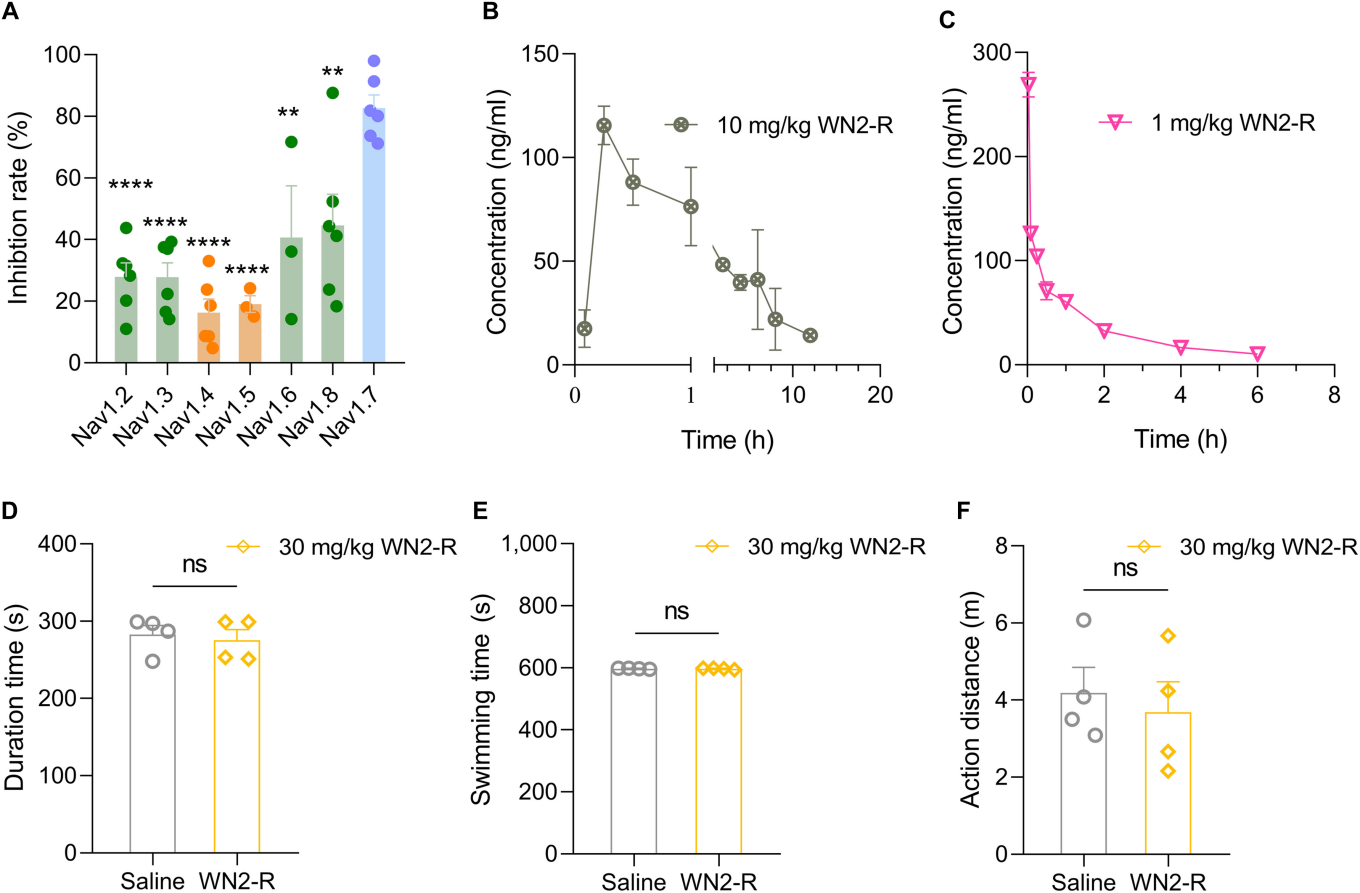
Positive subtype selectivity, drug safety, and bioavailability of WN2-R. (A) Under 1 μM, the inhibition rate of WN2-R on subtypes other than Na_v_1.7 was very low, indicating that WN2-R was a selective inhibitor. (B and C) Time course of concentration of mice: (B) under p.o. conditions (10 mg/kg); (C) under i.v. conditions (1 mg/kg). (D to F) The motor ability of WN2-R group mice was not affected during the rotarod test, forced-swim test, and open field test (*n* = 4) (**P < 0.01; ****P < 0.0001; ns, not significantly different).

VGSC inhibitors required credible subtype selectivity to void side effects caused by binding to nontarget subtypes. At a concentration of 1 μM, the inhibitory rates of WN2-R on hNa_v_1.2, hNa_v_1.3, hNa_v_1.4, hNa_v_1.5, mNa_v_1.6, and hNa_v_1.8 were determined (*n* = 3 to 6, Fig. 7A). The result showed that the activity of WN2-R on other subtypes was much lower than that on Na_v_1.7, with particularly low inhibitory rates on Na_v_1.4 (16.3%) and Na_v_1.5 (19.1%). This suggested that WN2-R had minimal side effect on mice exercise ability and low cardiac toxicity. To further evaluate the drug safety profile, high-dose acute toxicity experiments were conducted. Mice were administered a high dose of 30 mg/kg WN2-R through i.p. administration. The rotarod test, forced-swim test, and open field test were used to explore the effect of high-dose WN2-R on the motor ability of mice (*n* = 4). The rotarod test evaluated the drug's effect on motor coordination, the forced-swim test examined its impact on the overall motor system, and the open field test assessed the drug's influence on the motor and nervous systems [21–23]. The results indicated that mice in the experimental groups survived normally and displayed no notable changes in motor function compared to the saline group (Fig. 7D to F). This suggested that, even at a high dose of 30 mg/kg, WN2-R had no adverse effects on the motor abilities of mice, initially demonstrating its excellent drug safety profile.

To evaluate the pharmaceutical properties of WN2-R, a preliminary pharmacokinetic study was conducted in mice to examine the in vivo pharmacokinetic profile following both oral (p.o.) and intravenous (i.v.) administrations, along with the determination of bioavailability (Table 2). Under p.o. conditions, WN2-R was rapidly absorbed, with drug detection occurring within 5 min. The *T*_max_ was achieved at 15 min, followed by a gradual decrease in plasma concentration over time, approaching the detection limit by 12 h post-dosing (Fig. 7B). Calculation revealed a half-life (*t*_1/2β_) of 3.46 ± 0.50 h and a mean residence time (MRT_(0-*t*)_) of 5.66 ± 0.33 h. The *C*_max_ was 138.93 ± 25.39 ng/ml, and AUC_(0-*t*)_ was 541.93 ± 98.47 h·ng/ml. For the i.v. study, the *C*_max_ was detected at 2 min, reaching 269.02 ± 11.94 ng/ml. Similar to the p.o. study, the plasma concentration decreased gradually over time, approaching the quantitation limit at 6 to 8 h post-dosing (Fig. 7C). The *t*_1/2β_ and MRT_(0-*t*)_ for WN2-R were 4.47 ± 0.96 h and 3.21 ± 0.28 h, respectively, with an AUC_(0-*t*)_ of 267.03 ± 29.69 h·ng/ml. Finally, based on the AUC_(0-*t*)_ values from both administrations, the bioavailability (*F*) of WN2-R was calculated to be 20.29%. Compared with the positive control drug PF-05089771 (*F* = 27%), WN2-R exhibited a favorable bioavailability, suggesting its potential for further oral drug development [10]. Besides, these results also indicated that the testing time of the mice experimental model fell within an acceptable range. In conclusion, WN2-R exhibited good subtype selectivity, as well as positive drug safety and bioavailability, making it a strong candidate as a novel Na_v_1.7-selective inhibitor.

**Table 2.** Pharmacokinetic parameters of WN2-R in mice for the p.o. and i.v. routes

Parameters^a^	p.o. (10 mg/kg)	i.v. (1 mg/kg)
*t*_1/2_ (h)	3.46 ± 0.50	4.47 ± 0.96
*T*_max_ (h)	0.25	0.033
*C*_max_ (ng/ml)	138.93 ± 25.39	269.02 ± 11.94
AUC_(0-*t*)_ (h∙ng/ml)	541.93 ± 98.47	267.03 ± 29.69
AUC_(0-∞)_ (h∙ng/ml)	546.21 ± 98.12	271.32 ± 30.84
MRT_(0-*t*)_ (h)	5.66 ± 0.33	3.21 ± 0.28
MRT_(0-∞)_ (h)	5.87 ± 0.40	3.56 ± 0.44
*V* (ml/kg)	-	23,580.98 ± 2,679.45
CL (ml/h/kg)	-	3,717.70 ± 423.10
*F* (%)	20.29	-

^a^ *t*_1/2_: The time required for the plasma concentration of a drug to decrease by half; *T*_max_: The time it took for a drug to reach its maximum plasma concentration (*C*_max_); *C*_max_: The highest concentration of the drug in plasma; AUC_(0-*t*)_: The area from dosing time (0) to the last measured time point (*t*); AUC_(0-∞)_: The total area from dosing time (0) to infinity, which included extrapolation beyond the last time point; MRT_(0-*t*)_: From dosing to the last time point; MRT_(0-∞)_: From dosing to infinite time; *V*: The hypothetical volume required to distribute the drug uniformly at the same concentration as in plasma; CL: The volume of plasma cleared of the drug per unit time; F: Bioavailability.

## Discussion

The design of the new generation of selective VGSC inhibitors was a continuous focus in the development of novel analgesic drugs. In this study, we discovered that compound WN2 functioned as a highly active inhibitor of Na_v_1.7, targeting the VSDIV pocket. By combining computational analysis with experimental methods, we demonstrated that the R chirality was the preferred conformation of the compound. In numerous experiments utilizing acute pain, chronic pain, and neuropathic pain models in mice, WN2-R exhibited remarkable analgesic effects, with many data points surpassing the positive control drug PF-05089771. Furthermore, we determined the selectivity of WN2-R and conducted acute toxicity experiments and pharmacokinetic investigations. These results were consistent with our expectations, indicating that WN2-R held great potential for drug development. Consequently, our work contributed a novel Na_v_1.7-selective inhibitor with an entirely new structure, opening new avenues for fresh perspectives in the development of novel analgesic drugs.

**Fig. 8.**
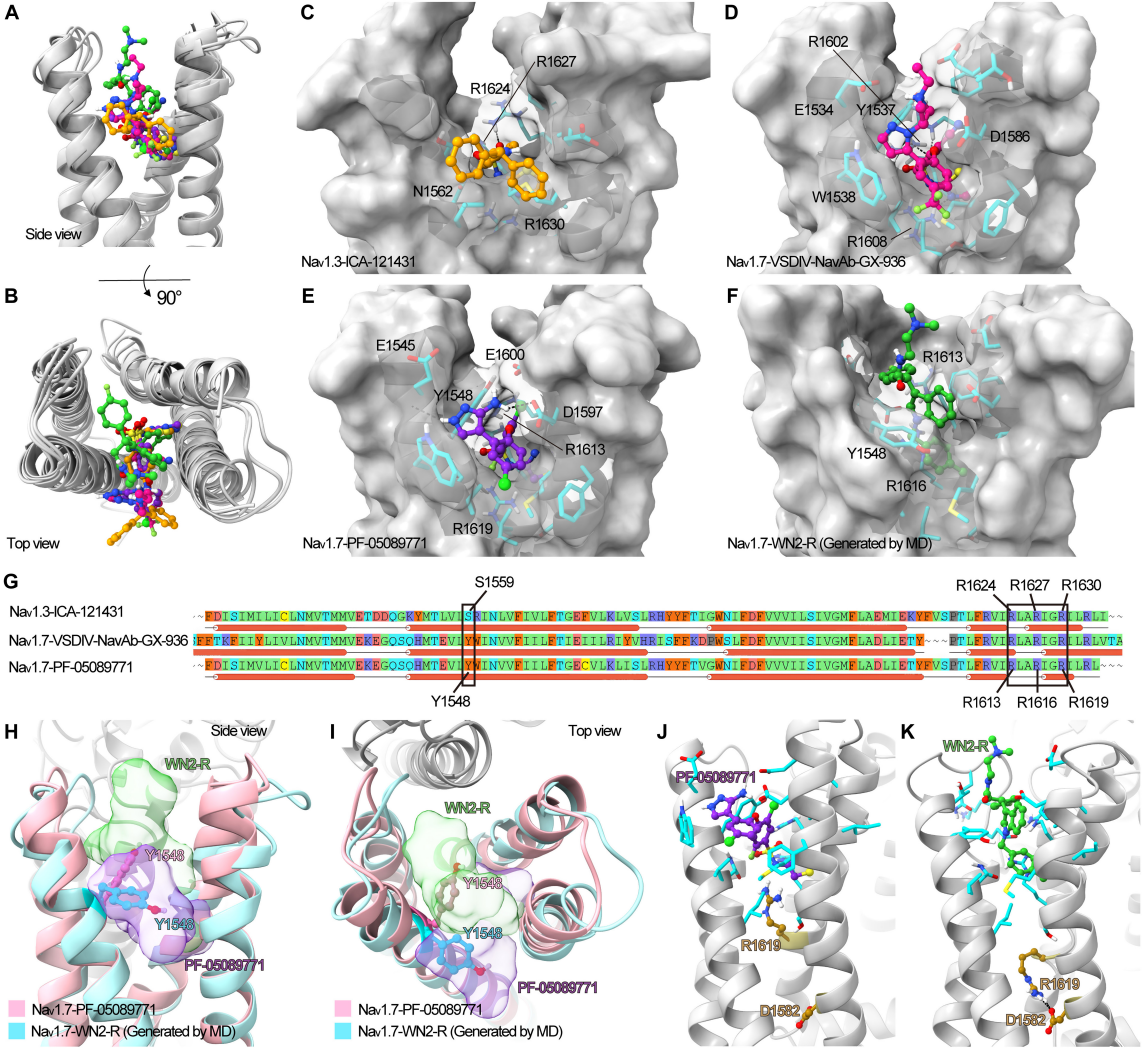
Comparison of the binding conformations between WN2-R and aryl sulfonamide ligands in the VSDIV pocket. (A and B) The side view and top view of the binding conformations, indicating the unique binding conformation of WN2-R. (C to F) The binding conformations and key residues. The ligands (ICA-121431: orange; GX-936: dark pink; PF-05089771: purple; WN2-R: green) were shown as stick–ball models. The receptor surfaces were displayed to better illustrate the differences of the VSDIV pocket upon the binding of different ligands. Polar interactions were represented by black dashed lines. (G) Comparison of sequences in VSDIV pockets. (H and I) The comparison of Y1548 (represented by the stick–ball model) upon the binding of WN2-R or PF-05089771. The complexes of VSDIV ligand (the MD-generated complex of VSDIV-WN2-R and the crystal structure) were aligned based on backbone atoms. The ligand (WN2-R or PF-05089771) was shown as transparent surface. (J and K) Comparison of R1619 (represented by the stick–ball model) orientation difference upon the binding of PF-05089771 and WN2-R.

As mentioned, the binding modes in the VSDIV pocket of all existing aryl sulfonamide antagonists were highly conservative [13]. We wondered whether WN2-R adopted a similar binding mode as aryl sulfonamides. Therefore, the clustered representative binding conformation in 4 MD simulations of WN2-R was taken for comparison with complexes Na_v_1.7-VSDIV-Na_v_Ab-GX-936 (PDB code: 5EK0) and Na_v_1.7-PF-05089771 (PDB code: 8I5G [24]). The complex Na_v_1.3-ICA-121431 (PDB code: 7W7F [25]) was also taken to compare the binding pocket difference among Na_v_s subtypes. The 4 complexes were aligned based on VSDIV atoms, and interestingly, there were clear differences in the spatial position occupied by WN2-R and the other 3 ligands. From the side view (Fig. 8A), when compared to 3 others, WN2-R took up marked more space longitudinally, but the center of mass of WN2-R was not as deep into VSDIV as 3 others, since the 2-(dimethyl amino) ethyl portion extended away from the interior of VSDIV. From the top view (Fig. 8B), WN2-R occupied the region in the middle of the 4 α-helices of VSDIV, whereas the other 3 ligands were sandwiched between the 2 helices and nearly half of them were located on the outer surface of VSDIV. These spatial positional differences had also been confirmed by the study of Kschonsak et al. [26] The results indicated that both aryl sulfonamide and acyl sulfonamide inhibitors exhibited a tendency to shift outward from the region between the 4 α-helices of VSDIV. To compare in detail, local interactions and the shape of induced binding pockets are demonstrated in Fig. 8C to F. In terms of interaction types, ICA-121431, GX-936, and PF-05089771 all showed obvious polar interactions with VSDIV, while no polar interaction was found in maintaining the binding of WN2-R. In the complex Na_v_1.3-ICA-121431, the ligand was anchored primarily by 2 arginines R1624 and R1630 through polar interactions with its sulfonamide moiety (Fig. 8C). A similar situation was observed for 2 aryl sulfonamide antagonists. In the complex Na_v_1.7-VSDIV-Na_v_Ab-GX-936, strong polar interactions were present between the sulfonamide portion of the ligand and 2 arginines R1602 and R1608, while E1534 and D1586 might form salt bridges with the cyclobutylammonium portion of the ligand (Fig. 8D). Correspondingly, R1613 and R1619 provided polar interactions for the sulfonamide portion of PF-05089771, while D1597 and E1600 acted as H-bond acceptors to stabilize the 3-aminopyrazole of the ligand (Fig. 8E). Based on Fig. 8A and B, the spatial positions of the sulfonamide portion were highly consistent among aryl sulfonamide antagonists. These indicated that the sequential conserved residues Na_v_1.3-R1624/Na_v_1.7-R1613 and Na_v_1.3-R1630/Na_v_1.7-R1619 (Fig. 8G) might be crucial for the binding of the Na_v_s-VSDIV antagonists containing the sulfonamide structure, while extra polar interactions might exist between aryl sulfonamide antagonists and residues at the opening of Na_v_1.7-VSDIV. By contrast, WN2-R showed a quite different interaction pattern, as no important polar interaction was observed for this ligand, indicating that hydrophobic and cation–π conjugation should be the major interactions (Fig. 8F).

On the other hand, the VSDIV binding pocket induced by WN2-R was completely different from that of the other 3 ligands. In the complex Na_v_1.3-ICA-121431, a shallow binding pocket was induced by the ligand (Fig. 8C), while in the complexes Na_v_1.7-VSDIV-Na_v_Ab-GX-936 and Na_v_1.7-PF-05089771, the side chain of Y1537/Y1548 was squeezed into the interior of VSDIV by the ligand, occupying the region between the 4 helices together with R1602/R1613, and the marginal region of VSDIV was occupied by the ligand (Fig. 8D and E). In the case of Na_v_1.7-WN2-R, the side chain of Y1548 was oriented away from the center of VSDIV, wrapping the ligand in the middle along with the remaining residues. From Fig. 8F, it could be seen that the toluene moiety of WN2-R penetrated deep into the hydrophobic cavity inside VSDIV. Although induced to very different conformations (Fig. 8H and I), Y1548 of Na_v_1.7 provided a Π–Π conjugate interaction for both aryl sulfonamide antagonists and WN2-R, and this residue corresponded in sequence to S1559 of Na_v_1.3, which indicated that this residue might be a key residue for designing selective Na_v_1.7 antagonists. R1619 of Na_v_1.7 was another residue that had a very different conformation upon the binding of aryl sulfonamide antagonists or WN2-R. When the former bound Na_v_1.7, the side chain of R1619 faced up and was involved in the polar interaction with the ligand. The representative conformation obtained by our MD simulations revealed that the side chain of this residue turned to face down, forming a salt bridge with D1582 (86.8% of the conformations, Fig. 8J and K). The impact of this difference on VSDIV of Na_v_1.7 needed to be further discussed in future studies.

Currently, most Na_v_1.7 inhibitors that have reached clinical trials have been discontinued. Thus, what potential benefits does WN2-R bring compared to these clinical candidates? Our study highlights several key advantages of WN2-R. Firstly, regarding its molecular structure, WN2-R diverges from the aryl sulfonamide scaffold, which commonly leads to high affinity for plasma proteins [27], a pitfall for many clinical drugs. The unique structure of WN2-R allowed it to avoid this risk, enhancing its delivery to the site of action. Secondly, in animal models encompassing acute, chronic inflammatory pain, and neuropathic pain, WN2-R demonstrated superior efficacy compared to the positive control drug PF-05089771. Notably, WN2-R markedly outperformed PF-05089771 in the neuropathic pain model, highlighting its potential for treating nerve injury-related pain. For clinical translation, WN2-R not only offered broad-spectrum pain-relieving potential but also presented unique advantage in treating nerve injury pain, providing a novel approach to analgesic drug development. Furthermore, different from the aryl sulfonamide ligands, the binding of WN2-R is primarily driven by induced fit, suggesting its distinct subtype selectivity. While high selectivity (over 1,000-fold) has been reported for aryl sulfonamides, excessively narrow targeting may overlook other signaling pathways, potentially leading to suboptimal outcomes. According to our previous research results, there might have been compensatory mechanisms among Na_v_1.7, Na_v_1.8, and Na_v_1.9, with Na_v_1.8 potentially playing a more important role in APs [28]. Therefore, an excessively high selectivity for Na_v_1.7 might not have yielded desirable effects, and the selectivity range of WN2-R might be more reasonable. As a result, we believe that WN2-R, with its ability to avoid high-affinity risks with plasma proteins, broad analgesic applicability, unique efficacy in neuropathic pain, and balanced subtype selectivity make it a promising candidate for clinical translation and novel analgesic development. Overall, our study still had many limitations, and further in-depth explorations were needed to reveal additional novel mechanisms. We will continue to optimize the structure and properties of WN2-R, exploring the direction of next-generation analgesic drug development.

## Materials and Methods

### Discovery of WN1 and WN2

The crystal structure of Na_v_1.7-VSDIV-Na_v_Ab (PDB code: 5EK0 [29]), which contained the key ligand (GX-936) bound to the VSDIV pocket and provided a critical reference for determining the binding site of the VSDIV pocket, was used as the template for SBVS. The protein structure was prepared through the Protein Preparation Wizard [30] in Schrödinger2018 [31]. The docking grid box, encompassing the VSDIV pocket, was generated via the Receptor Grid Generation wizard. The ChemDiv (https://www.chemdiv.com/) and Specs (https://www.specs.net/) databases were downloaded and processed by the LigPrep module, and the generated ligands were docked into the prepared protein structure using Glide with the SP precision. The top 5,000 compounds, ranked based on their docking scores, were selected for further analysis. Then, these compounds were filtered using the REOS mode in Canvas3.7 to remove molecules with poor druglike properties. The remaining molecules were then clustered (threshold = 0.85) based on their MACCS fingerprints. Furthermore, we calculated the binding free energies for the remaining compounds using the Prime MM/GBSA approach [32–35]. Candidate molecules from each database were aggregated and analyzed, and compounds with duplicated structures were removed. Based on the results, 14 compounds were ultimately purchased for further biological assays.

After obtaining WN1, its skeleton was chosen as the structural scaffold, and the analogs sharing the same scaffold were retrieved from the ChemDiv database. Moreover, the 2D structure of WN1 was used for similarity search (threshold = 0.7) based on the MACCS fingerprints to find additional structures. The compounds retrieved from both protocols were compared and analyzed. Finally, 39 compounds were purchased for further bioassays.

### MD simulations

The cryo-electron microscopy structures of Na_v_1.7 (PDB code: 6J8J [36]) were downloaded from the PDB. The protein structure was refined using the Protein Preparation Wizard in Schrödinger2018. This process involved removing irrelevant components, adding missing loops potentially relevant to VSDIV, and adding missing side chains and heavy atoms. To generate the initial complex of WN2 with Na_v_1.7, the IFD protocol of Schrodinger2018 was utilized to dock the ligand into the supposed binding site. In this protocol, the side chains within the binding grid were permitted to undergo induced-fit adjustment upon ligand binding.

The prepared protein structure was subsequently assigned to the CHARMM-GUI server [37], adding palmitoyl oleyl phosphatidyl choline (POPC, ~320 per layer), water, and ions (Na^+^ and Cl^−^), to produce a combined system with approximately ~360,000 atoms. The partial atom charges of the docked WN2 were calculated using the restrained electrostatic potential (RESP) protocol, which involved fitting the electrostatic potentials computed at the HF/6-31G level in Gaussian16 [38]. The force field parameters for the ligands were assigned through the Antechamber module in AMBER18 [39]. The ff14SB force field [40], General Amber Force field [41], and Lipid17 force field were used for the proteins, ligands, and POPC molecules, respectively. For each system, all components were seamlessly merged using the *tleap* program in AMBER18 [42].

Afterwards, the *pmemd* program in AMBER18 was used to execute the minimization, MD heating, and MD equilibration. Initially, a restraint force of 10.0 kcal/(mol·Å^2^) was applied to the protein, WN2, and membrane atoms, while the solvent and ions were allowed to be optimized by 5,000 cycles of steepest descent and 5,000 cycles of conjugate gradient minimizations. Secondly, the protein backbone and WN2 atoms were restrained by an elastic force of 4.0 kcal/(mol·Å^2^), and the receptor side chains underwent optimization with 5,000 cycles of steepest descent and 5,000 cycles of conjugate gradient minimizations. Finally, all atoms were optimized by 5,000 cycles of steepest descent and 5,000 cycles of conjugate gradient minimizations without any restraint. Each optimized system was gradually heated from 0 to 310 K over 0.1 ns with the protein, WN2, and membrane atoms restrained by an elastic force of 8.0 kcal/(mol·Å^2^). Then, in the equilibration stage, the system was allowed to relax in the NPT ensemble (*P* = 1 bar, *T* = 310 K). The elastic force of 4.0 kcal/(mol·Å^2^) was added to the protein, WN2, and membrane atoms for 0.5 ns, then reduced to act solely on the receptor backbone atoms for another 0.5 ns [2.0 kcal/(mol·Å^2^)], and finally completely removed to relax the system for 2.0 ns. For each system, the production run was conducted for 500 ns, replicated 4 times with varying initial atom velocities. The Langevin temperature equilibration scheme, with a collision frequency of 2.0 ps^–1^, was used to control the temperature. The long-range electrostatic interactions under the periodic boundary condition were handled by the particle mesh Ewald (PME) algorithm with a cutoff of 8 Å for the real-space interactions [43]. All covalent bonds involving hydrogen atoms were constrained using the SHAKE algorithm, with a time step of 2 fs [44]. The ligand RMSF was calculated by the *cpptraj* module in the AMBER18 package, and the average binding free energy between WN2 and the receptor for each trajectory was calculated by the VAD-MM/GBSA method [45,46].

### Electrophysiology

#### Cell culture and transfection

Human embryonic kidney (HEK) 293T (ATCC CRL-3216) and ND7/23 cells (National Collection of Authenticated Cell Cultures SCSP-5026) were cultured under standard conditions (5% CO_2_, 37 °C) in Dulbecco’s modified Eagle’s medium (DMEM) containing 10% fetal bovine serum and 1% penicillin-streptomycin solution(all from Gibco, Thermo Fisher Scientific, Waltham, MA, USA). When the cells reached 70% to 90% confluence, human Na_v_1.2 (hNa_v_1.2), human Na_v_1.3 (hNa_v_1.3), human Na_v_1.4 (hNa_v_1.4), human Na_v_1.5 (hNa_v_1.5), and mouse Na_v_1.6 (mNa_v_1.6) channel plasmids were cotransfected with enhanced green fluorescent protein (eGFP) into HEK293T cells. Additionally, plasmids expressing β1 and β2 subunits tagged with eGFP were cotransfected with the human Na_v_1.7 (hNa_v_1.7) channel into HEK293T cells. Human Na_v_1.8 (hNa_v_1.8) was transiently transfected into ND7/23 cells, along with eGFP, using Lipofectamine 2000 (Invitrogen), adhering strictly to the manufacturer’s protocol. For manual patch-clamp experiments, the cells were plated onto glass coverslips and used within 20 to 48 h of transfection [47,48].

#### DRG isolation and culture

DRG isolation and culture were performed according to our previous report [49]. Briefly, C57BL/6 mice (weight 18 to 20 g, 6 to 8 weeks) were euthanized via cervical dislocation under anesthesia. Lumbar DRGs were isolated from the L4 to L5 segments of the lumbar spinal cord. The DRG neurons were subsequently dissociated through enzymatic digestion with collagenase (0.32 mg/ml) and trypsin (0.15 mg/ml) at 37 °C for 30 min, with intermittent shaking. After centrifugation at 800 rpm for 5 min at room temperature, the cells were seeded onto poly-L-lysine-coated coverslips and cultured in DMEM (Gibco) supplemented with 10% fetal bovine serum (Gibco). The cells were incubated at 37 °C with 5% CO_2_ in a humidified incubator for 3 h before whole-cell patch-clamp recording. Please note that male and female mice in half were used in each experiment.

#### Whole-cell patch-clamp electrophysiology

Whole-cell patch-clamp recordings were conducted at 25 ± 2 °C, utilizing an EPC 10USB patch-clamp amplifier (HEKA Elektronik, Lambrecht, Germany). Pipettes, with access resistance between 2.0 and 3.0 MΩ, were pulled from borosilicate glass capillary tubes using a 2-step vertical microelectrode puller (PC-10, Narishige Group, Tokyo, Japan). Voltage-clamp recordings were obtained with PatchMaster software v2 × 73 (HEKA Elektronik) 5 min after establishing whole-cell configuration, sampled at 20 kHz, and filtered at 5 kHz. To minimize voltage errors, 80% series resistance compensation was applied. Pipettes were filled with 140 mM CsF, 10 mM NaCl, 1 mM EGTA, and 10 mM HEPES (pH=7.4, adjusted with CsOH). The external solution contained 140 mM NaCl, 2 mM CaCl_2_, 1 mM MgCl_2_, 5 mM KCl, 10 mM HEPES, and 10 mM glucose (pH=7.4, adjusted with NaOH). IC_50_ values were determined in HEK293T or ND7/23 cell lines by voltage clamping at −80 mV, followed by stepping to the V_1/2_ of inactivation for 8 s to facilitate compound binding.

For recording Na_v_ currents of DRG neurons, the bath solution contains (in mM) 30 NaCl, 1 MgCl_2_, 1.8 CaCl_2_, 5 CsCl, 5 KCl, 25 D-glucose, 5 HEPES, 0.1 CdCl_2,_ and TEA-Cl (pH 7.4 with NaOH), and the pipette solution contains (in mM) 135 CsCl, 10 NaCl, and 5 HEPES (pH 7.4 with CsOH). The Na_v_ current program is recorded following the above method. For recording the voltage-gated K^+^ currents in DRG neurons, the bath solution contained (in mM) 130 choline chloride, 5 KCl, 2 MgCl_2_, 2 CaCl_2_, 10 HEPES, and 10 D-glucose, (pH 7.3 with Tris), and the pipette solution contained (in mM) 120 KCl, 20 NMG, 10 EGTA, and 10 HEPES (pH 7.3 with KOH). Cells were elicited by 300 ms depolarization potential to +30 mV from a holding voltage of −80 mV. For recording the voltage-gated Ca^2+^ currents in DRG neurons, the bath solution contained (in mM) 140 TEA-Cl, 2.5 CsCl, 0.6 MgCl_2_, 5 BaCl_2_, 10 HEPES, and 10 D-glucose (pH 7.3 with CsOH), and the pipette solution contained (in mM) CsMeSO_4_ KCl, 11 EGTA, 2 Mg-ATP, and 10 HEPES (pH 7.3 with CsOH). Cells were elicited by 300 ms depolarization potential to 0 mV from the holding voltage of −90 mV. For current-clamp recording, the extracellular solution contained (in mM) 140 NaCl, 3 KCl, 2 CaCl_2_, 2 MgCl_2_, and 10 HEPES (pH 7.3 with NaOH); the pipette solution contained (in mM) 140 KCl, 0.5 EGTA, 5 HEPES, and 2 Mg-ATP (pH 7.3 with KOH). To record DRG neurons’ APs, the mode was switched to current clamp and the cell was clamped at 0 pA. Stimulations ranging from 0 to 90 pA were applied for 1 s with a 5-s sweep (1 Hz).

### Mice pain models

#### Animals

Healthy ICR or C57BL/6 mice (18 to 22 g) were obtained from the Experimental Animal Center of Hunan SLac-kinda. These mice were housed in a controlled environment with a constant temperature of 24 °C and humidity ranging from 50% to 60% under a 12-h light/dark cycle and provided with free access to laboratory-standard food and water.

#### Study approval

All animal experiments were performed in accordance with the Guidelines for Laboratory Animal Research established by Hunan Normal University and were approved by the Institutional Animal Care and Use Committee of the College of Medicine in Hunan Normal University.

#### Acetic acid-induced writhing test

Three male and 3 female ICR mice were used in the acetic acid-induced writhing test. Mice were preadministered with either WN2-R (5 mg/kg i.g., 1 mg/kg i.m.), PF05089771 (5 mg/kg i.g., 1 mg/kg i.m.), or saline 15 min before i.p. injection of 0.2 ml of acetic acid (0.8%, V/V). Each mouse was then placed in an individual open polyvinyl cage (30 cm × 40 cm × 30 cm). The number of writhing responses, characterized by a wave of abdominal muscle contraction followed by hind limb extension, was recorded for 30 min after acetic acid injection. These responses served as an indication of nociceptive intensity. The percentage of inhibition was calculated using the following equation: Inhibition% = (Writhing Number_vehicle_ – Writhing Number_drug_)/Writhing Number_vehicle_ × 100% [15,47].

#### Formalin-induced inflammation pain model

To induce the pain response, 20 μl of formalin (5%, w/v) was injected subcutaneously into the right hind paw of ICR mice (3 male and 3 female). Prior to the formalin injection, mice were preadministered with WN2-R (5 mg/kg i.g., 1 mg/kg i.m.), PF05089771 (5 mg/kg i.g., 1 mg/kg i.m.), or saline 30 min beforehand. The duration of licking/biting the injected paw was recorded every 5 min, commencing immediately after the formalin injection and continuing for 40 min. Phase I and II in this assay corresponded to the nociceptive response observed between 0 and 5 min and between 10 and 40 min, respectively [17,21].

#### CFA-induced inflammatory pain model

The mechanical allodynia of ICR mice (2 male and 2 female) was assessed prior to model construction using von Frey fibers according to the up–down method [18]. Mice with paw withdraw thresholds (PWTs) remarkably deviating from the mean value were excluded from further experiments. Subsequently, 15 μl of CFA was unilaterally injected into the intraplantar surface of the right hind paw of each mice. After 24 h, the PWTs of the mice were assessed. During the i.g. administration of drugs or saline, PWTs were measured 0, 0.5, 1, 1.5, 2, and 2.5 h post drugs/saline administration. Similarly, during i.m. administration, PWTs were measured at 0, 0.5, 1, 1.5, 2, 2.5, and 3 h post drugs/saline administration.

#### SNI model

To elucidate the time course of pain hypersensitivity induced by SNI, the behavioral responses (mechanical testing with von Frey fibers) were measured before surgery (baseline value) in C57 mice (5 female). Then, the mice underwent SNI surgery, which involved ligation and axotomy of the common peroneal and tibial nerves, while sparing the sural nerve. Sham-operated mice served as controls, with their nerves remaining intact. To confirm the presence of pain hypersensitivity in the SNI group, behavioral testing was carried out 14 days after SNI in each animal. Post drugs/saline administration, PWTs were measured at 0, 0.5, 1, 1.5, 2, 2.5, and 3 h for the i.g. route, and at 0, 0.5, 1, 2, 3, and 4 h for the i.m. route [19].

### Safety evaluation

#### Rotarod test

ICR mice were trained for 4 days at a consistent speed of 20 rpm for 10 min daily. No further experiments were carried out in mice whose running time significantly deviated from the mean. On the 4th day, the mice were randomly divided into 2 groups (saline group and 30 mg/kg WN2-R group). Both saline and WN2-R were administered i.p., and behavioral tests were conducted 30 min after the administration. The total running time of animals within 300 s was recorded using a constant speed rotating rod test [22].

#### Forced-swim test

ICR mice were placed in an 80 cm × 40 cm pool. Mice with swim times significantly differing from the average were excluded from the experiment. The remaining mice were trained to swim in the pool for 3 days, with each session lasting 10 min [21]. The mice were randomly divided into the saline group and 30 mg/kg WN2-R group, and 2 groups were administered i.p. and behavioral tests were performed 30 min after the administration.

#### Open field test

The testing area for the mice typically consisted of a 42 cm × 42 cm × 42 cm polyvinyl chloride (PVC) box, with a camera used to monitor their movement within the central and peripheral zones of the box. The movement within 8 min inside the mice box was recorded, and the movement path of the mice within 4 to 6 min was analyzed, and the movement distance of the mice was recorded [23]. The mice were randomly divided into the saline group and 30 mg/kg WN2-R group, and 2 groups were administered i.p. and behavioral tests were performed 30 min after the administration.

### Pharmacokinetics of mice

#### Pharmacokinetic study of WN2-R

The pharmacokinetic and bioavailability of WN2-R were examined in ICR mice following both p.o. and i.v. administrations. All animal experiment procedures were carried out in accordance with the animal experiment procedures promulgated by the State Science and Technology Commission of China and Animal Care and Use Committee of Institute of Materia Medica, Chinese Academy of Medical Sciences (registry no. 00008517). Male ICR mice (*n* = 12), weighing 20 ± 2 g, were obtained from Beijing Vital River Experimental Animal Co., Ltd. (Beijing, China). Before the experiment, the mice were kept under standard conditions, including a 12-h light/dark cycle, adequate ventilation, a temperature of 25 ± 2 °C, and 60% relative humidity. WN2-R, suspended in physiological saline, was orally administrated to mice at 10 mg/kg after an overnight fast. In addition, the mice were injected via caudal vein with WN2-R dissolved in 20% HP-β-CD at 1 mg/kg. Blood samples were collected from the retro-orbital venous plexus to ice-cooled EP tube containing 0.5% heparin sodium at 0.033 (i.v. only), 0.083, 0.25, 0.5, 1, 2, 4, 6, 8, 12, and 24 h after dosing. Plasma was obtained by centrifugation of blood at 8,000 rpm for 5 min. Subsequently, 20 μl of plasma sample was mixed with 140 μl of acetonitrile, vortexed for 30 s, and centrifuged twice at 14,000 rpm for 5 min. The resulting supernatant (2 μl) was analyzed by the liquid chromatography–tandem mass spectrometry (LC-MS/MS) method [50].

#### LC-MS/MS analysis

The LC-MS/MS system consisted of Thermo TSQ Access Max mass and Vanquish liquid chromatograph (Thermo Scientific, USA). Zorbax-SB C_18_ column (2.1 × 100 mm, 3.5 μm Agilent USA) was used in the analysis. The mobile phase consisted of water containing 0.1% formic acid (Phase A) and acetonitrile (Phase B) at a flow rate of 0.25 ml/min with an operation temperature of 35 °C. A gradient elution system was used as follows: 40% B at 0 to 1 min, 90% B at 1 to 4 min, 90% B down to 40% B at 4 to 5 min, and 40% B at 5 to 7 min. The automatic sampler was maintained at 15 °C throughout the analysis. For the mass spectrometry analysis, positive ion scanning in selected reaction monitor mode was applied to monitor the transition of *m/z* 458.2 to 191.9 for the determination of WN2-R. Besides, other instrument parameters, including ion spray voltage, capillary temperature, sheath gas, auxiliary gas, and collision energy, were optimized to enhance the analysis of WN2-R.

#### Data analysis

EXCEL and PRISM8.0 were used for data and chart preparation. The Winnolin professional software was employed for the pharmacokinetic parameter calculation. Bioavailability was calculated using the following formula: *F* (%) = (*D*_i.v._ × AUC_p.o._)/(*D*_p.o._ × AUC_i.v._) × 100%.

### Chemical synthesis and chiral separation

#### Chemical synthesis of WN2

The general synthetic sequence for WN2 is outlined in Fig. S1. 1*H*-Indole (**1**) was used as the starting material. Intermediate **3** was obtained through nucleophilic substitution. Subsequently, intermediate **4** was generated via a 3-component reaction involving Meldrum’s acid, 4-fluorobenzaldehyde, and intermediate **3**. The hydrolysis and decarboxylation of intermediate **4** yielded **5**, which was then condensed to form the compound WN2 [51–53].

#### Enantioseparation of WN2 by preparative HPLC

The enantioseparation of WN2 was conducted by using a preparative chiral HPLC under the following conditions: column: CHIRALPAK IG (IG00CE-XL022); injection: 20 μl; mobile phase: ACN/DEA = 100/0.1 (v/v); flow rate: 1.0 ml/min; wavelength: UV 254 nm; HPLC equipment: Shimadzu LC-20AT CP-HPLC-07. The optical isomers of WN2 were automatically collected. Organic solvent was subsequently removed using a rotary evaporator at 35 °C, yielding both (R)-WN2 and (S)-WN2.

#### ECD data acquisition and calculation

The ECD spectrum was recorded with a standard sensitivity of 200 mdeg, a data pitch of 0.5 nm, and a bandwidth of 1 nm. Continuous scanning mode was employed with a scanning speed of 100 nm/min, a response time of 1 s, and a scanning wavelength range of 190 to 400 nm using quartz cuvettes with a pathlength of 0.1 or 1 cm. The collected data were corrected using the baseline of the corresponding solvent and smoothed using a means-movement equation. A systematic conformational search was performed using molecular mechanics with the MMFF94 force field in the MOE software package, and 27 conformers within an energy window of 10 kcal/mol were taken as the initial conformations. Density functional theory (DFT) and its time-dependent variant (TDDFT) calculations were carried out using Gaussian16RevB.01. The meta-hybrid functional with Grimme’s D term (ωB97XD) and the TZVP basis set were used, and the lowest 80 electronic transitions were set. A solvation model using density was applied to simulate the effects of the solvent. Finally, the Gibbs free energies were calculated under conditions of 298.15 K and 1 atm pressure to identify the most populated conformers. The Boltzmann-averaged ECD spectra were obtained with σ as 0.45 eV using the SpecDis1.71 software.

## Data Availability

According to the Creative Commons Attribution-NonCommercial 4.0 International (CC BY-NC 4.0) license, all data in this study can be viewed and downloaded for free. However, these data are strictly prohibited from being used for any commercial purposes.
