# Supplementary material for: Discovery of Novel Nav1.7-Selective Inhibitors with the 1H-Indole-3-Propionamide Scaffold for Effective Pain Relief
Source: Research (Wash D C). 2025 Jan 29;8:0599. doi: 10.34133/research.0599 (PMC11775380; doi:10.34133/research.0599)
Supplement: Supplementary 1 — Tables S1 and S2 Figs. S1 and S2 [file research.0599.f1.docx]

**Supplementary Information**

**Table S1. The chemical structures and inhibitory activity of the 14 compounds from structure-based virtual screening.**

| **ID** | **SMILES** | **Inhibitory activity (10 μM)** |
| --- | --- | --- |
| A1 | C1CC2=C(C1)N(C(=O)N=C2SCC(=O)NCC3=CC=C(C=C3)F)CC4=CN=CC=C4 | -0.67% ± 5.03% |
| A2 | COC1=C(C=C(C=C1)S(=O)(=O)NC2=CC3=C(C=C2)N(CCC3)C(=O)C4=CC=CC=C4)OC | 36.56% ± 0.43% |
| A3 | C1=CC=C(C=C1)CCNC(=O)CC(C2=CC(=CC=C2)OCC3=CC=CC=C3)C4=CN(C5=CC=CC=C54)CC6=CC=C(C=C6)F | 9.09% ± 3.43% |
| A4 | C1CC1N(CC(=O)NC2=NC(=CN2C3=CC=CC=C3)C4=CC=CC=C4)C(=O)C5=CC=C(C=C5)Cl | 76.38% ± 3.77% |
| A5 | COCCNC(=O)CC(C1=CC(=C(C=C1)OCC2=CC=CC=C2)OC)C3=CN(C4=CC=CC=C43)CC5=CC=C(C=C5)F | 3.57% ± 1.05% |
| A6 | C1=CC=C2C(=C1)C(=CN2CC3=CC=C(C=C3)F)C(CC(=O)NCCC4=CC=CC=N4)C5=CC=C(C=C5)C(F)(F)F | 73.21% ± 1.08% |
| A7 | FC(C=C1)=CC=C1CN2C=C(C(CC(NCC3COC(C=CC=C4)=C4O3)=O)C5=CC(Cl)=CC=C5)C6=CC=CC=C62 | 57.96% ± 5.30% |
| A8 | COC1=CC(=C(C=C1)N2CC(CC2=O)C(=O)NC3CCCC4=C3NC5=CC=CC=C45)OC | 12.06% ± 3.76% |
| A9 | C1=CC=C(C=C1)OC2=CC=C(C=C2)C3=C(N=C(N3)C4=CC=C(C=C4)Br)C5=CC=C(C=C5)OC6=CC=CC=C6 | 4.44% ± 1.46% |
| A10 | C1=CC=C2C(=C1)C(OC2=O)NC3=CC=C(C=C3)S(=O)(=O)NC4=CC=CC=N4 | 2.74% ± 2.11% |
| A11 | CS(=O)(=O)C1=CC2=C(C=C1)N=C(S2)NC(=O)NC3=CC(=C(C=C3)Cl)Cl | 4.91% ± 2.01% |
| A12 | CC(=O)N1CCC2=C(C1)C(=C3C(=C(SC3=N2)C(=O)NC4=CC=CC(=C4)C#N)N)C(F)(F)F | 9.32% ± 0.44% |
| A13 | CCOC1=CC=C(C=C1)C2N(C(=NN2C3=CC(=C(C=C3)Cl)Cl)C(=O)C)C4=CC=C(C=C4)N5CCN(CC5)C(=O)C | 15.85% ± 2.76% |
| A14 | C1=CC=C(C(=C1)C(F)(F)F)NC(=O)C2=CC3=C(C=C2)C(=O)N(C3=O)CC4=CN=CC=C4 | 2.68% ± 1.57% |

**Table S2. The chemical structures and inhibitory activity of the 39 compounds from computational structure optimization.**

| **ID** | **SMILES** | **Inhibitory activity (10 μM)** |
| --- | --- | --- |
| B1 | C1=CC=C(C=C1)CN2C=C(C3=CC=CC=C32)C(CC(=O)NCCC4=C(C=C(C=C4)Cl)Cl)C5=CC=C(C=C5)F | 6.13% ± 2.52% |
| B2 | C1=CC=C(C=C1)CN2C=C(C3=CC=CC=C32)C(CC(=O)NCC4=CC=CC=N4)C5=CC=C(C=C5)F | 11.47% ± 15.08% |
| B3 | CC1=CC=C(C=C1)C(CC(=O)NCCC2=CC=CC=N2)C3=CN(C4=CC=CC=C43)CC5=CC=C(C=C5)OC | 14.24% ± 13.40% |
| B4 | C1=CC=C2C(=C1)C(=CN2CC3=CC=C(C=C3)F)C(CC(=O)N)C4=CC=C(C=C4)C(F)(F)F | 13.45% ± 2.49% |
| B5 | CC1=CC(=CC=C1)C(CC(=O)NCCC2=CC=CC=C2)C3=CN(C4=CC=CC=C43)CC5=CC=C(C=C5)F | 2.93% ± 4.10% |
| B6 | CC1=CC(=CC=C1)C(CC(=O)NCCC(C)C)C2=CN(C3=CC=CC=C32)CC4=CC=C(C=C4)F | 7.88% ± 11.80% |
| B7 | CCCCNC(=O)CC(C1=CC=CC(=C1)C)C2=CN(C3=CC=CC=C32)CC4=CC=C(C=C4)F | 1.84% ± 1.33% |
| B8 | C1CC1NC(=O)CC(C2=CC=C(C=C2)F)C3=CN(C4=CC=CC=C43)CC5=CC=CC=C5 | 13.59% ± 1.99% |
| B9 | CC1=CC=C(C=C1)CN2C=C(C3=CC=CC=C32)C(CC(=O)NC4CCCCC4)C5=CC=C(C=C5)F | 6.85% ± 2.97% |
| B10 | CC1=CC=C(C=C1)CN2C=C(C3=CC=CC=C32)C(CC(=O)NCCN(C)C)C4=CC=C(C=C4)F | 91.53% ± 6.26% |
| B11 | C1CCN(C1)CCNC(=O)CC(C2=CC=C(C=C2)C(F)(F)F)C3=CN(C4=CC=CC=C43)CC5=CC=CC=C5 | 7.97% ± 6.90% |
| B12 | CC1=CC(=C(C=C1)F)C(CC(=O)NCCC2=CC=CC=N2)C3=CN(C4=CC=CC=C43)CC5=CC=C(C=C5)OC | 4.98% ± 7.35% |
| B13 | C1=CC=C2C(=C1)C(=CN2CC3=CC=C(C=C3)F)C(CC(=O)NCCC4=CC=CC=N4)C5=CC(=CC=C5)OC6=CC=C(C=C6)Cl | 6.28% ± 8.11% |
| B14 | CN1C=C(C2=CC=CC=C21)C(CC(=O)NCCC3=CC=CC=N3)C4=CC(=CC=C4)C(F)(F)F | 24.80% ± 16.85% |
| B15 | CCOC1=CC=C(C=C1)C(CC(=O)NCCC2=CC=CC=N2)C3=CN(C4=CC=CC=C43)CC5=CC=C(C=C5)OC | 20.51% ± 12.71% |
| B16 | C1=CC=C(C=C1)COC2=CC=C(C=C2)C(CC(=O)NCCC3=CC=CC=N3)C4=CN(C5=CC=CC=C54)CC6=CC=C(C=C6)F | 4.16% ± 3.58% |
| B17 | COC1=C(C=C(C=C1)C(CC(=O)NCCC2=CC=CC=N2)C3=CN(C4=CC=CC=C43)CC5=CC=C(C=C5)F)OC | 34.88% ± 27.01% |
| B18 | CC1=CC=C(C=C1)CN2C=C(C3=CC=CC=C32)C(CC(=O)NCCC4=CC=CC=N4)C5=CC=C(C=C5)F | 13.72% ± 16.14% |
| B19 | C1=CC=C2C(=C1)C(=CN2CC3=CC=C(C=C3)F)C(CC(=O)NCCC4=CC=CC=N4)C5=CC=C(C=C5)F | 22.87% ± 10.86% |
| B20 | COC1=CC=C(C=C1)C(CC(=O)NCCC2=CC=CC=N2)C3=CN(C4=CC=CC=C43)CC5=CC=C(C=C5)F | 37.53% ± 11.34% |
| B21 | CCN1CCN(CC1)C(=O)CC(C2=CC=C(C=C2)C(F)(F)F)C3=CN(C4=CC=CC=C43)CC5=CC=C(C=C5)F | 71.39% ± 8.10% |
| B22 | COC1=CC(=CC(=C1)C(CC(=O)NCCC2=CC=CC=N2)C3=CN(C4=CC=CC=C43)CC5=CC=CC=C5)OC | 45.04% ± 13.56% |
| B23 | C1=CC=C2C(=C1)C(=CN2CC3=CC=C(C=C3)F)C(CC(=O)NCC4=CC=NC=C4)C5=CC=C(C=C5)C(F)(F)F | 18.19% ± 19.13% |
| B24 | C1COCCN1CCNC(=O)CC(C2=CC=C(C=C2)C(F)(F)F)C3=CN(C4=CC=CC=C43)CC5=CC=C(C=C5)F | 15.99% ± 13.86% |
| B25 | C1CCN(C1)C(=O)CC(C2=CC=C(C=C2)C(F)(F)F)C3=CN(C4=CC=CC=C43)CC5=CC=C(C=C5)F | 23.09% ± 17.76% |
| B26 | COC1=CC(=CC(=C1)C(CC(=O)NCCC2=CC=CC=N2)C3=CN(C4=CC=CC=C43)CC5=CC=C(C=C5)F)OC | 26.10% ± 10.37% |
| B27 | C1=CC=C2C(=C1)C(=CN2CC3=CC=C(C=C3)F)C(CC(=O)NCCC4=CC=CC=N4)C5=CC(=CC=C5)Cl | 15.98% ± 15.89% |
| B28 | C1=CC=C(C=C1)OC2=CC=CC(=C2)C(CC(=O)NCCC3=CC=CC=N3)C4=CN(C5=CC=CC=C54)CC6=CC=C(C=C6)F | 29.24% ± 26.64% |
| B29 | COC1=CC=C(C=C1)CN2C=C(C3=CC=CC=C32)C(CC(=O)NCCC4=CC=CC=N4)C5=CC=C(C=C5)OC | 9.42% ± 6.82% |
| B30 | COC1=CC=C(C=C1)CN2C=C(C3=CC=CC=C32)C(CC(=O)NCCC4=CC=CC=N4)C5=CC(=CC=C5)Cl | 8.62% ± 7.68% |
| B31 | C1CN(CCC1N(C2=CC3=C(C=C2)OCO3)S(=O)(=O)C4=CC=C(C=C4)F)CC5=CN=CC=C5 | 16.04% ± 5.49% |
| B32 | C1=CC=C(C=C1)CN2C=C(C3=CC=CC=C32)C(CC(=O)NCCC4=CC=CC=N4)C5=CC=C(C=C5)C(F)(F)F | 16.62% ± 18.34% |
| B33 | C1=CC=C2C(=C1)C(=CN2CC3=CC=C(C=C3)F)C(CC(=O)NCCC4=CC=CC=N4)C5=CC(=CC=C5)C(F)(F)F | 19.75% ± 17.35% |
| B34 | CC(C)CNC(=O)CC(C1=CC=C(C=C1)C(F)(F)F)C2=CN(C3=CC=CC=C32)CC4=CC=CC=C4 | 13.39% ± 6.95% |
| B35 | C1=CC=C2C(=C1)C(=CN2CC3=CC=C(C=C3)F)C(CC(=O)NCC4=CC=NC=C4)C5=CC(=CC=C5)C(F)(F)F | 15.62% ± 9.88% |
| B36 | C1=CC=C(C=C1)CN2C=C(C3=CC=CC=C32)C(CC(=O)NCC4=CC=NC=C4)C5=CC=C(C=C5)C(F)(F)F | 17.20% ± 2.86% |
| B37 | (C)C(CC(=O)NCCC1=CC=CC=N1)C2=CN(C3=CC=CC=C32)CC4=CC=CC=C4 | 19.13% ± 16.30% |
| B38 | CN1C=C(C2=CC=CC=C21)C(CC(=O)NCCC3=CC=CC=N3)C4=CC=C(C=C4)C(F)(F)F | 13.90% ± 3.84% |
| B39 | C1CC(N(C1)C(=O)C2=CSC(=N2)NC3=CC=C(C=C3)F)C(=O)N4CCOCC4 | 16.67% ± 16.16% |

**Figure S1. Synthesis of N-substituted-1*H*-indole-3-amide WN2.** Conditions: (a) KOH, DMF, rt, overnight, 60%; (b) Meldrum’s acid, 4-fluorobenzaldehyde, L-proline, rt, 18 h, 78%; (c) DMF, H_2_O, 100 ℃, 4 h, 83%; (d) N,N-dimethylethylenediamine, HATU, DIPEA, DMF, rt, 3 h, 64%.

**General Considerations:**

Unless otherwise stated, all commercially available starting materials were of reagent grade and solvents were of analytical grade and used without further purification. All air-sensitive reactions were magnetically stirred under an argon atmosphere. ^1^H NMR and ^13^C NMR spectra were recorded on Mercury-400 and Mercury-500 spectrometers at room temperature. Chemical shifts (δ) are reported in ppm downfield from internal TMS standards and J values are given in Hz. LC–MS (ESI) and HRMS (ESI): M/Z data measured on a Thermo Exactive Orbitrap plus spectrometer. Flash column chromatography was performed on a Biotage Isolera one.

**1-(4-methylbenzyl)-1*H*-indole (3)**:

Indole (469 mg, 4 mmol) was dissolved in DMF (6 mL) at room temperature under a nitrogen atmosphere, and KOH (269 mg, 4.8 mmol) was then added into the reaction mixture and stirred at room temperature. After all the KOH were dissolved, benzyl bromide (740 mg, 4 mmol) was added dropwisely and stirred overnight. 10 mL of water was added to the mixture and stirred for 30 min. The mixture was then extracted with DCM and the organic layer was washed with water twice. The organics were dried over Na_2_SO_4_, concentrated, and purified by column chromatography over silica gel (PE) to afford title compound (530 mg, 60% yield) as a colorless oily liquid: LC–MS (ESI) *m/z* 222.18 [M + H]^+^;^1^H NMR (400 MHz, CDCl_3_) δ 7.67 (d, *J* = 7.7 Hz, 1H), 7.31 (d, *J* = 8.2 Hz, 1 H), 7.19 (t, *J* = 6.9 Hz, 1 H), 7.15–7.08 (m, 4 H), 7.03 (d, *J* = 8.1 Hz, 2 H), 6.56 (d, *J* = 3.2 Hz, 1 H), 5.30 (s, 2 H), 2.33 (s, 3 H).

**5-((4-fluorophenyl)(1-(4-methylbenzyl)-1*H*-indol-3-yl)methyl)-2,2-dimethyl-1,3-dioxane-4,6-dione (4):**

To a mixture of Meldrum’s acid (144 mg, 1 mmol), 4-Fluorobenzaldehyde (124 mg, 1 mmol) and compound **3** (221 mg, 1 mmol) in acetonitrile 5 mL was added. L-proline (6 mg 0.05 mmol) was also added. The mixture was stirred for 18 h at room temperature. The solvent was removed under reduced pressure and the residue was purified by column chromatography over silica gel (PE/EA = 85:15) to afford title compound (370 mg, 78% yield) as a white solid: LC–MS (ESI) *m/z* 472.17 [M + H]^+^; ^1^H NMR (400 MHz, CDCl_3_) δ 7.43–7.35 (m, 3 H), 7.33 (s, 1 H), 7.25 (d, *J* = 6.4 Hz, 1 H), 7.15 (t, *J* = 7.8 Hz, 1 H), 7.11 (d, *J* = 7.8 Hz, 2 H), 7.07–7.00 (m, 3 H), 6.95 (t, *J* = 8.5 Hz, 2 H), 5.65 (s, 1 H), 5.29 (d, *J* = 5.0 Hz, 2 H), 4.26 (d, *J* = 2.6 Hz, 1 H), 2.31 (s, 3 H), 1.72 (s, 3 H), 1.45 (s, 3 H).

**3-(4-fluorophenyl)-3-(1-(4-methylbenzyl)-1*H*-indol-3-yl)propanoic acid (5):**

Compound **4** (471 mg, 1 mmol) was dissolved in mixture of DMF (10 mL) and water (1 mL). Resulted solution was stirred and heated in oil bath at 100 ℃ for 4 h. After completion of the reaction, solvent was removed under reduced pressure and DCM was added. The organic layer were dried over Na_2_SO_4_, concentrated, and purified by column chromatography over silica gel (PE/EA = 80:20) to afford title compound (530 mg, 83% yield) as a white solid: LC–MS (ESI) *m/z* 388.24 [M + H]^+^;^1^H NMR (500 MHz, DMSO-*d*_6_) δ 12.14 (s, 1 H), 7.48 (s, 1 H), 7.41–7.32 (m, 4 H), 7.11–7.00 (m, 7 H), 6.90 (t, *J* = 7.4 Hz, 1 H), 5.31 (s, 2 H), 4.65 (t, *J* = 7.9 Hz, 1 H), 3.07 (dd, *J* = 15.5, 7.6 Hz, 1 H), 2.95 (dd, *J* = 15.5, 8.2 Hz, 1 H), 2.24 (s, 3 H).

***N*-(2-(dimethylamino)ethyl)-3-(4-fluorophenyl)-3-(1-(4-methylbenzyl)-1*H*-indol-3-yl)propenamide (WN2):**

Compound **5** (387 mg, 1 mmol), *N*,*N*-dimethylethylenediamine (106 mg, 1.2 mmol), HATU (570 mg, 1.5 mmol), and DIPEA (646 mg, 5 mmol) were stirred in DMF (5 mL) at room temperature. The reaction mixture was stirred for 3 h. After completion of the reaction, solvent was removed under reduced pressure and EA was added. The solution was washed with water twice, dried over Na_2_SO_4_, concentrated, and purified by column chromatography over silica gel (DCM/MeOH = 96:4) to afford title compound (293 mg, 64% yield) as a white solid: HRMS (ESI) m/z calcd for C_29_H_33_ON_3_F [M + H]^+^ 458.26022; found 458.25986; ^1^H NMR (500 MHz, DMSO-*d*_6_) δ 7.79 (t, *J* = 5.7 Hz, 1 H), 7.42 (s, 1 H), 7.35 (d, *J* = 8.2 Hz, 1 H), 7.32–7.26 (m, 3 H), 7.13–6.99 (m, 7 H), 6.89 (t, *J* = 7.1 Hz, 1 H), 5.31 (s, 2 H), 4.67 (t, *J* = 7.9 Hz, 1 H), 3.03 (ddt, *J* = 24.1, 13.4, 6.9 Hz, 2 H), 2.88 (dd, *J* = 14.1, 7.6 Hz, 1 H), 2.74 (dd, *J* = 14.0, 8.1 Hz, 1 H), 2.24 (s, 3 H), 2.13 (q, *J* = 6.7 Hz, 2 H), 2.08 (s, 6 H). ^13^C NMR (126 MHz, DMSO-*D*_6_) δ 170.25, 161.52, 159.60, 140.93, 136.45, 136.22, 135.28, 129.37, 129.30, 129.05, 126.96, 125.74, 121.25, 119.06, 118.56, 117.32, 114.84, 114.67, 110.10, 58.04, 48.84, 44.96, 42.28, 38.06, 36.48, 20.64.


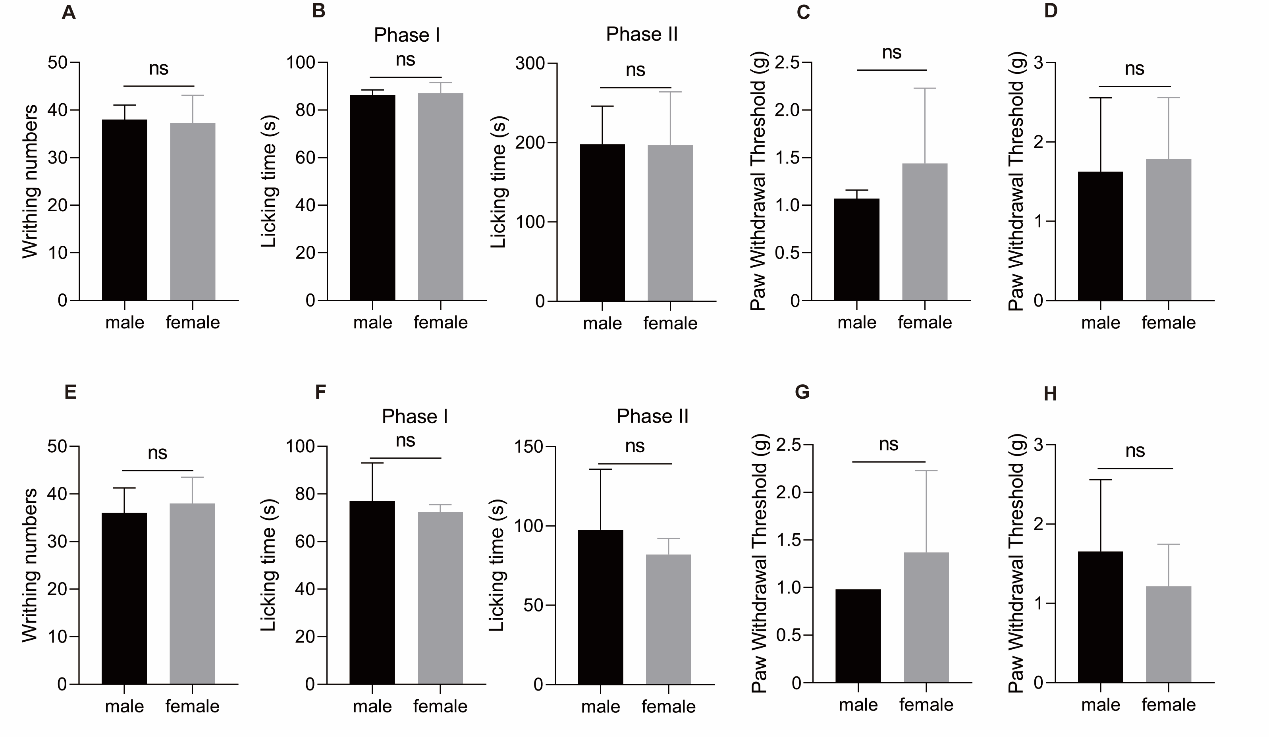


**Figure S2. Sex-dependent analysis of the analgesic effects of WN2-R in the mouse pain model.** (A) and (E) Acetic-acid induced writhing test (i.g. and i.p.) with WN2-R (male = 3, female = 3). (B) and (F) Formalin induced inflammation pain model (i.g. and i.m.): Statistics of the total licking times in phase I (left) and phase II (right) for each group (male = 3, female = 3). (C) and (G) CFA induced chronic pain model (i.g. and i.m.): Statistics of the PWTs 1.5 h post drug application male = 2, female = 2). (D) and (K), SNI pain model (i.g. and i.m.): Statistics of the PWTs at 1 h post drug application ((male = 2, female = 3). (*p < 0.05; **p < 0.01; ***p < 0.001; ns, not significantly different).
